# LINTUL-Cassava-NPK: A simulation model for nutrient-limited cassava growth

**DOI:** 10.1016/j.fcr.2022.108488

**Published:** 2022-05-15

**Authors:** J.G. Adiele, A.G.T. Schut, K.S. Ezui, K.E. Giller

**Affiliations:** a Plant Production Systems Group, Wageningen University, P.O. Box 430, 6700 AK Wageningen, The Netherlands; b National Root Crops Research Institute, Umudike, KM 8 Ikot Ekpene Road, P.M.B 7006, Umuahia, Abia State, Nigeria; c African Plant Nutrition Institute, ICIPE Campus, Duduville – Kasarani, Thika Road, Nairobi, Kenya

**Keywords:** Nutrient uptake rates, Balanced nutrition, Simulation model, Potassium response, Nutrient equivalents

## Abstract

A solid understanding of the dynamics of plant nutrient requirements and uptake from the soil is needed to provide robust fertilizer recommendations, timing of applications and nutrient use efficiency. Our objective was to develop and test the ability of the crop model LINTUL-Cassava-NPK to simulate biomass growth and yield of cassava under nutrient-limited conditions. We used experimental data from six fields located in three different agro-ecologies in Nigeria: Rainforest Zone– Ogoja and Ikom (Cross River), Rainforest Transition Zone – Ekpoma (Edo) and Guinea Savanna Zone – Otukpo (Benue) over two consecutive growing seasons from 2016 to 2018. Nutrient stress in the model was implemented by combining N, P and K nutrition indices (NI) to account for the interaction of multiple nutrient limitations for crop growth. Nutrient uptake was determined by balancing demand and supply of nutrient equivalents. We parameterized and calibrated the model using observations from an experiment conducted under optimal growing conditions in Edo during the 2016 planting season. The model was then tested with data from experiments conducted in the 2017 season in Edo, Cross River and Benue. The model captured the uptake patterns of N, P and K well. Uptakes of N, P and K, and storage root yield were predicted with a small root mean squared error of 5.1 g N m^−2^, 0.8 g P m^−2^, 3.3 g K m^−2^ and 308 g DM roots m^−2^, with an *R*^2^ of 0.7 – 0.8 for linear relationships between simulated and observed values. The time course of development of nutrient-limited yield of green leaves, stems and storage roots were simulated reasonably well. In general, the model responded aptly to both nutrient omissions and varying amounts of NPK. These findings increase our understanding of nutrient limitations and N, P and K interactions on cassava growth and yield. The model provided insight into surplus amounts of nutrients in the soil at the end of the season and, specifically, the need to balance the supply of N and K for cassava. To our knowledge, this is the first tested cassava process-based model that includes the three macro-nutrients.

## 1 Introduction

Cassava (*Manihot esculenta* Crantz) is a major staple food crop in the tropics and is a feed stock for industrial starch processing and associated rural development (Rahman and Awerije, 2016, FAO, 2018). Cassava is becoming increasingly important as a commercial crop and now cultivated on large scale to meet the growing demand. The current average fresh storage root yield in smallholder farmers’ fields in sub-Saharan Africa (SSA) is estimated at only 7.2 t ha^−1^ (FAOSTAT, 2020), while yields in research trials varied between 8.6 and 55.5 t ha^−1^ of fresh storage root yield (Ezui, 2017; Fermont et al., 2007; Eke-Okoro and Njoku, 2012). Sustainable intensification of cassava production is necessary for food security in SSA, especially given projected population growth (UNDESA, 2018). Cassava can grow in relatively poor soils with a low soil fertility and moderate acidity under erratic rainfall conditions (Howeler, 2017). Yet, it has a very high yield potential when growth conditions and management are optimal, and can yield over 90 t ha^−1^ of fresh storage roots (Adiele et al., 2020, Byju and Suja, 2020; Cock et al., 1979). Insufficient supply of N, P and K restricts the productivity of the crop. Understanding the dynamics of nutrient requirements and the impact of uptake limitations of cassava during the growth cycle enables prediction of cassava yields under nutrient limited conditions and may provide insight into best management practices to improve nutrient use efficiency. The nutrient contents of organs of the cassava plant vary during the growth cycle, but concentrations in the leaves fluctuate within narrow ranges (Cock and Connor, 2021; Adiele et al., 2021a). Typically, plants dilute nutrients over time as carbon accumulates (Lemaire, 2012). Nutrients are translocated from one organ to another depending on the sink strength (Shibu et al., 2010), and whether they are mobile in the phloem (Eichert and Fernández, 2012). Although N, P and K have specific roles in plants (Hawkesford et al., 2012), their limitations impact average growth rates in a more or less linear fashion compared with when nutrient supply is balanced (Janssen et al., 1990). A crop’s demand for nutrients at any given time is presumed to be determined by the difference between the current nutrient content in the tissue and a possible maximum content (Shibu et al., 2010).

Nutrient uptake rates decrease during drought for several reasons: the inability to transport nutrients, including reduction of nutrient supply through mineralization (Bista et al., 2018), partly compensated by higher concentrations in soil water and stronger diffusion. Severe drought strongly decreases nutrient demand and uptake rates from soil; the impact on plant growth depends on the intensity and duration of drought stress and the developmental stage of the crop (El-Sharkawy, 2007, Mithra et al., 2013). Demand later in the growing season may also be reduced due to stomatal responses when vapour deficits are large (Vongcharoen et al., 2018). A decrease in nutrient uptake will result in reduced growth rates when nutrients are deficient, which affects canopy cover and light interception.

Nitrogen, P and K deficiencies affect cassava growth and yield in three major ways: reduction of leaf growth rates and hence light interception; through reduction of the photosynthetic rate of leaves, and changes in the sink strength and partitioning of assimilates to the different organs (Cock and El-Sharkawy, 1988, Pellet and El-Sharkawy, 1993). Nitrogen availability affects photosynthesis through its impact on chlorophyll content, leaf area development and photosynthetic efficiency (Sangakkara and Wijesinghe, 2014; Cock and Connor, 2021). Phosphorus availability strongly affects leaf growth and LAI, photosynthetic activity, the number of storage roots formed and hence yield, although responses varied among cultivars (Pellet and El-Sharkawy, 1993). Potassium has numerous functions in crop growth (Eichert and Fernández, 2012, Engels et al., 2012) and influences leaf appearance rates and leaf size (Jordan-Meille and Pellerin, 2004, Engels et al., 2012). Wasonga et al. (2020) observed that in drought-stressed young cassava plants, increasing K concentration increased net photosynthesis by increasing leaf water potential and turgor, which in turn increased stomatal conductance. Potassium also plays a major role in the transfer of sucrose and storage of starch in cassava storage roots (Cock and Connor, 2021). Ezui et al. (2017) observed that K applications increased radiation use efficiency (*RUE*) in cassava, while N increased the amount of light intercepted. N, P and K deficiency is mainly associated with a reduction of leaf growth rates which limits leaf area and hence light interception (Mwamba et al., 2021), with a small decrease in net photosynthesis due to source or sink limitations and radiation use efficiency, particularly in the case of K, when crops are drought stressed.

Knowledge of nutrient (N, P and K) demand and uptake patterns under deficient conditions in cassava (Adiele et al., 2021a) can be used to develop a crop simulation model. After testing, such a model may be used for many purposes, including to generate crop responses for series of years to characterize cassava growth and nutrient uptake, provide location-specific fertilizer recommendations, and to extrapolate from the studied area to other areas where less detailed information is available. There are few dynamic crop growth models for the simulation of nutrient-limited growth in crops. The Light INTerception and UTilization (LINTUL3) model for rice (Shibu et al., 2010) and Agricultural Production Systems Simulator (APSIM) for pearl millet (Akponikpè et al., 2010) models were developed primarily to simulate the crops responses to N supply. APSIM (P) was further developed to simulate the effects of P on common bean and maize (Delve et al., 2009). The simulation model of cassava (SIMCAS) simulates N and K uptake and effects on cassava (Mithra et al., 2013), assuming a proportional reduction in growth rate due to limitations of N or K. The modelling approach of Shibu et al. (2010) in LINTUL3 incorporated the effects of nitrogen shortage on crop growth through growth reduction factors, similar to the approach used to simulate water stress in LINTUL2 for water-limited yield. A major breakthrough in crop growth modelling is needed to simulate NPK interactions and the relationships between soil available nutrients and crop demand over time and space. With an in-depth understanding of plant nutrient demand and uptake under favourable and adverse conditions, it becomes possible to provide robust and more effective fertilizer recommendations that are in line with the 4 R (right source at the right amount, right time and right place) nutrient stewardship principles (Luar et al., 2018). In this study, our objectives were to develop and test a dynamic model for cassava that simulates growth and predicts yields under N, P and K limited conditions. To this end, we further developed the previously tested LINTUL-Cassava model (Ezui et al., 2018, Adiele et al., 2021b) and included processes to estimate nutrient uptake and effects of NPK limitations on crop growth rates. The purpose of this model is to predict nutrient uptake and yield based on a few parameters that summarize more detailed processes (van Ittersum et al., 2003).

## 2 Materials and methods

### 2.1 Model description

LINTUL–Cassava model was developed to simulate potential and water-limited production. The modelling concept focuses on the main processes that are needed to predict storage root yields, using Monteith’s RUE concept (Monteith, 1977) with a descriptive component for assimilate partitioning as function of thermal time. The crop’s development as simulated by the model includes three development stages: (1) planting to sprouting, (2) sprouting to first branching and (3) first branching to harvest, as described in Ezui et al. (2018). The LINTUL-Cassava-NPK model describes N, P and K demand and uptake by the crop in detail. The effect of the nutrients deficiency was expressed through a combined growth reduction factor, called nutrient nutrition index (NPKI). The following main processes in cassava growth and development are described in the LINTUL-Cassava-NPK model: (1) soil supply of NPK, (2) plant nutrient demand, (3) nutrient uptake and partitioning, (4) nutrient redistribution and (5) nutrient stress. Limitations by N, P or K further increased the partitioning to the fine roots, in the same manner as water deficiency. Treatments with more or less balanced NPK supply did not differ in the proportional distribution of biomass over leaves, stems and storage organs (Figs. S2 to S4 in supplementary materials). The proportional changes in stem and storage roots observed at final harvest were approximately proportional to K supply (Fig. S2). However, some effects of N, P or K limitation on partitioning were observed. For example, P limitation strongly affected yields but did not affect the measured leaf biomass at final harvest (Fig. S4). For balanced treatments, the proportion of biomass in storage roots, stems and leaves at harvest were 0.62, 0.34 and 0.04 respectively. Imbalanced nutrition with severe limitations of P or K in treatments without P or K, combined with large amounts of NK or NP, changed these proportions to 0.54, 0.38 and 0.08 for 0 P treatments and to 0.54, 0.42 and 0.04 for 0 K treatments (Fig. S1). These effects of imbalanced nutrition on storage root yields are expected to be relatively small, for example in Edo (2016) total storage root biomass for treatments without P or K ranged from 24 to 32 t DM ha^−1^ resulting in a model error for yield of at most 2.6 t DM ha^−1^. Such extreme imbalances in nutrient supply are not expected on farm fields and the effects of nutrient limitations on partitioning were therefore not included in the LINTUL-Cassava-NPK model.

### 2.2 Soil supply of N, P and K

In the model, available N, P and K (g m^−2^) for crop uptake is from two sources: nutrients present in the soil profile during the growing season and N, P and K applied as fertilizers. Whilst N (and to a lesser extent P) are mineralized from soil organic matter, P and K availability depends on the equilibrium between so-called plant available pools (the quantity or Q factor) and the concentration in the soil solution (the intensity or I factor) (Marschner and Rengel, 2012). Nutrients in the soil are modelled with three states per nutrient, including the amount of plant available nutrient in the soil (*A*, g m^−2^), and total nutrient from soil pools (S, g m^−2^) and total available nutrients from fertilizer application (*F*, g m^−2^). It is assumed that 25% of total soil N, P and K supply was available at emergence, the remainder is assumed to become available at a constant rate, matching observed uptake patterns (Adiele et al., 2021a). The daily change in plant available nutrients (*RA*, g m^−2^ d^−1^) was determined as follows:

(1) $${\mathrm{RA}}_{N,P,K}=\frac{0.75\;\times \;S_{N,P,K}}{0.9\;\times \;\mathrm{SL}}+\left(R{\mathrm{ec}}_{N,P,K}\times {\mathrm{rf}}_{N,P,K}\times F_{N,P,K}\right)\times \mathrm{WLIMIT}-{\mathrm{RUptake}}_{N,P,K}$$

It is assumed that nutrients from the *S* pool (S is a combination of measured nutrient uptake in plots without nutrient applications and an additional measured uptake when other macro nutrients are supplied), becomes available in 0.9 of total season length (*SL*) in days. For fertilizers, the relative release rates (*rf*) are assumed constant, with *rf*_*N*_ = 0.1, *rf*_*P*_ = 0.01 and *rf*_*K*_ = 0.04 and *RUptake* is the daily uptake of nutrients. Under drought, soil water limitation (*WLIMIT*) affect crop growth, reduces nutrient uptake, including fertilizer release rates. *WLIMIT* was calculated as follows:

(2) $$\mathrm{WLIMIT}=\frac{\mathrm{TRANRF}}{K_{\mathrm{WATER}}+\mathrm{TRANRF}}$$

Where *TRANRF* is the transpiration reduction factor that reflects the drought stress of the crop, and *K*_*WATER*_ is a parameter that was set to a value of 0.2 (Table 1). *TRANRF* was defined as the ratio between actual and potential transpiration where actual transpiration is limited by soil water content, a crop specific transpiration coefficient and potential transpiration.

**Table 1:** Parameters related to N, P and K limited crop growth.

Parameter	Parameter description	Value	Units	Source of default values
*RDRNS*	Maximum relative death rate of the leaves due to nutrient stress	0.05	d^−1^	Estimated
*TSUM_NPKI*	Minimal *TSUM* before which nutrient limitations do not reduce growth rates	272	^0^C	Calibrated
*FR_MAX*	Optimal NPK concentration as fraction of maximum NPK concentration	0.8	–	Measured
*Rec*_*N*_	Maximum N recovery	0.75	–	Measured
*Rec*_*P*_	Maximum P recovery	0.28	–	Measured
*Rec*_*K*_	Maximum K recovery	0.7	–	Measured
*TC*_*NPK*_*T*	Time coefficient for NPK translocation	10	d	Estimated
*K*_*NI*_	K value in Monod relationship for *NPKI* (Eq. 11)	6.1	–	Calibrated
*KMAX*	Maximum value of K	4	–	Calibrated
*K_WATER_*	K value in Monod relationship with *TRANRF*	0.20	–	Estimated
*RNE*	Maximum nutrient equivalent uptake rate	0.012	d^−1^	Measured
*Soil N supply*	N uptake from control treatment	Site specific	kg N ha^−1^	Measured
*Soil P supply*	P uptake from control treatment	Site specific	kg P ha^−1^	Measured
*Soil K supply*	K uptake from control treatment	Site specific	kg K ha^−1^	Measured
*Extra N supply*	Extra N uptake from PK treatment	Site specific	kg N ha^−1^	Measured
*Extra P supply*	Extra P uptake from NK treatment	Site specific	kg P ha^−1^	Measured
*Extra K supply*	Extra K uptake from NP treatment	Site specific	kg K ha^−1^	Measured

### 2.3 Nutrients in the crop

For each plant organ, the model tracked changes in states for biomass and N, P and K uptakes. Minimum and maximum concentrations for each organ were provided as a function of temperature sum (*TSUM*), and were linearly interpolated to arrive at daily values. The *TSUM* is the physiological variable driving plant development (van Keulen and Seligman, 1987), and is determined as a function of the daily effective temperature after the planting date (Ezui et al., 2018). All organs are assumed to be at equivalent nutrition index values, i.e. at the same relative distance to minimum and maximum concentrations. Nutrients are translocated between organs with a short delay and a time coefficient of 10 days was set. Although reallocation of nutrients was modelled, it does not have an effect on crop growth. Nutrient uptake depended on total nutrient demand and availability of nutrients in the soil.

### 2.4 Crop nutrient translocation

Nutrient uptake was allocated to organs depending on the weight of organs. Nutrients in dying leaves are reallocated to stem (N) or storage roots (P and K). The total amounts of N, P and K lost with dying leaves were not known. Howeler (2012) showed that nutrient content in fallen leaves varied considerably by cultivar, environment and management, with an average of 26%, 14% and 10% of N, P and K of the whole plant nutrient uptake contained in the fallen leaves. We assumed however that the net nutrient loss of these fallen leaves is zero as a considerable amount of these nutrients will be recycled in the same growing season. This assumption was in our view reasonable, considering that the measured recovery of nutrients from fertilizers and nutrient supply from the soil was underestimated and did not include estimates of the amount of nutrients in dead leaves on the soil. These more detailed within-season recycling processes are not included in the model and therefore nutrient contents of dead leaves were set to zero.

### 2.5 Crop demand and uptake of N, P and K

The nutrient demand of the plant is calculated as the sum of the demand of each organ, computed as the difference between the actual and maximum amounts of nutrient in the plant organs. First, the actual and maximum amounts of nutrients in the plant (g m^−2^) were calculated from actual (*Ca*) or maximum concentrations (*Cm*) and weights (*W*) of leaves (*L*), stems (*ST*), storage organs (*SO*) and roots (*RT*):

(3) $${\mathrm{Act}}_{N,P,K}={\mathrm{CaL}}_{N,P,K}\times \mathrm{WL}+{\mathrm{CaST}}_{N,P,K}\times \mathrm{WST}+{\mathrm{CaSO}}_{N,P,K}\times \mathrm{WSO}+{\mathrm{CaRT}}_{N,P,K}\times \mathrm{WRT}$$

(4) $$M{\mathrm{ax}}_{N,P,K}={\mathrm{CmL}}_{N,P,K}\times \mathrm{WL}+{\mathrm{CmST}}_{N,P,K}\times \mathrm{WST}+{\mathrm{CmSO}}_{N,P,K}\times \mathrm{WSO}+{\mathrm{CmRT}}_{N,P,K}\times \mathrm{WRT}$$

Minimum and maximum concentrations of plant organs change as function of temperature sum. The measured values for minimum and maximum concentrations of N, P and K in leaves, stems and storage organs were provided in look-up tables and linearly interpolated to obtain daily values. These values were multiplied with weights of plant organ to obtain the total minimum (*Min*_N,P,K_) (Table S7) and maximum (*Max*_N,P,K_) amounts. The actual uptake of N, P and K (g m^−2^) is determined by crop demand and the plant available nutrients in the soil. We observed that plant uptake of one nutrient depends on the availability of other nutrients, a limited supply of one nutrient also resulted in reduced concentrations of the more abundantly supplied nutrients when compared to the maximum concentrations for a given weight of plant organs. To determine total nutrient demand and supply, both were therefore converted to nutrient equivalents (NE).

Nutrient equivalent demand (*NED*, g m^−2^) was determined as:

(5) $$\mathrm{NED}=\left({{\mathrm{Max}}_{N}-\mathrm{Act}}_{N}\right)+\frac{\left({{\mathrm{Max}}_{P}-\mathrm{Act}}_{P}\right)}{{\mathrm{Max}}_{P}}\times {\mathrm{Max}}_{N}+\frac{\left({{\mathrm{Max}}_{K}-\mathrm{Act}}_{K}\right)}{{\mathrm{Max}}_{K}}\times {\mathrm{Max}}_{N}$$

The nutrient equivalents in the soil (*NES*, g m^−2^) were computed as:

(6) $$\mathrm{NES}=A_{N}+\frac{A_{P}}{{\mathrm{Max}}_{P}}\times {\mathrm{Max}}_{N}+\frac{A_{K}}{{\mathrm{Max}}_{K}}\times {\mathrm{Max}}_{N}$$

The parameter *RNE* represents the maximum uptake rate of nutrient equivalents and its value was set to the observed maximum value of 0.012 d^−1^ reflecting the highest measured uptake rates of N, P and K, as provided in Adiele et al. (2021a).

Actual uptake rate of nutrient equivalent uptake is limited by either demand or supply:

(7) $$\frac{{\mathrm{dNE}}_{\mathrm{UP}}}{\mathrm{dt}}=\mathrm{RNE}\times \min(\mathrm{NES},\;\mathrm{NED})$$

The actual uptake rate (*UP*) of N, P and K is further limited by demand for these nutrients and uptake should not exceed total demand for a specific nutrient or maximum soil supply per time-step (*DELT*):

(8) $$\frac{{\mathrm{dUP}}_{N,P,K}}{\mathrm{dt}}=\min\left(\frac{{\mathrm{Max}}_{N,P,K}-{\mathrm{Act}}_{N,P,K}}{\mathrm{DELT}},\frac{A_{N,P,K}}{\mathrm{DELT}},\frac{{\mathrm{Max}}_{N,P,K}}{{\mathrm{Max}}_{N}}\times \frac{{\mathrm{dNE}}_{\mathrm{UP}}}{\mathrm{dt}}\right)$$

### 2.6 Redistribution of N, P and K

The rates of change in nutrient contents in leaves, fine roots, stems and storage organs are governed by redistribution from cuttings, uptake from soil and translocation between organs. In cassava, nutrients are redistributed to living plant organs from stem cuttings to form shoots at sprouting, from dying leaves to stem and storage roots at senescence, and from storage roots to form new leaves after dormancy (Alves, 2002, Howeler, 2012, Ezui et al., 2018, Adiele et al., 2021a). The N, P and K partitioning from cuttings followed assimilate partitioning governed by plant development and temperature sums. Partitioning of N, P and K taken up from soil to plant organs was proportional to biomass weight of plant organs. After drought, storage roots provide assimilates for new leaf formation. The associated transfer of NPK from storage roots to leaves was proportional to the reduction in storage root biomass. Nitrogen from dying leaves was transferred to stems, and P and K to storage roots (Adiele et al., 2021a). These processes result in imbalanced nutrient contents of organs. Nutrients in the plant were therefore translocated between plant organs based on their proportional demand with some delay. To this end, the transfer rates were computed by multiplying the difference between actual nutrient content and proportional demand of the organ with a relative redistribution rate (with a value of 0.1 d^−1^).

### 2.7 Nutrient stress

Crops undergo nutrient stress when plant nutrient concentrations are below critical values for maximum growth rates. Nutrition indices (*NI*) were defined for N, P and K and can range from 0 (acute shortage or deficiency of the nutrient) to 1 (nutrient sufficiency) status of the crop. Following Greenwood et al. (1986) and Angus and Moncur (1985), the nutrition indices (NI) were calculated as:

(9) $${\mathrm{NI}}_{N,P,K}=\frac{{\mathrm{Act}}_{N,P,K}-{\mathrm{Min}}_{N,P,K}}{{\mathrm{Opt}}_{N,P,K}-{\mathrm{Min}}_{N,P,K}}\;\mathrm{where}\;\mathrm{Act}\;\leq \mathrm{Opt}\;$$

Where *Act* reflects the actual amount of nutrients in the plant, *Min* the minimum amount and *Opt* the optimum amount needed for maximum growth rates. The optimum amount was computed as follows:

(10) $${\mathrm{Opt}}_{N,P,K}={\mathrm{Min}}_{N,P,K}+\mathrm{FR}\_\mathrm{MAX}\times \left({\mathrm{Max}}_{N,P,K}-{\mathrm{Min}}_{N,P,K}\right)$$

FR_MAX is a parameter which was set at a value of 0.8 (Table 1).

The three nutrition indices were multiplied to account for interaction effects:

(11) $$\left\{\begin{array}{c} \mathrm{NPKI}=\frac{c\times {\mathrm{NI}}_{N}\times {\mathrm{NI}}_{P}\times {\mathrm{NI}}_{K}}{K_{\mathrm{NI}}+{\mathrm{NI}}_{N}\times {\mathrm{NI}}_{P}\times {\mathrm{NI}}_{K}}\;\mathrm{when}\;K_{\mathrm{NI}}\leq \mathrm{KMAX}\;\\ \mathrm{NPKI}=1-\frac{c\times \left(1-{\mathrm{NI}}_{N}\times {\mathrm{NI}}_{P}\times {\mathrm{NI}}_{K}\right)}{K_{\mathrm{NI}}+\left(1-{\mathrm{NI}}_{N}\times {\mathrm{NI}}_{P}\times {\mathrm{NI}}_{K}\right)}\;\mathrm{when}\;K_{\mathrm{NI}}>\mathrm{KMAX} \end{array}\right\}$$

Where *c* equals *K*+ 1 to ensure that when there is no stress, the *NPKI* is also exactly 1, irrespective of the value of *K*_*NI*_. The values for *KMAX* (value of 4) and *K*_*NI*_ (value of 6.1) were calibrated (Table 1). These values mean that *NPKI* was more strongly reduced when multiple nutrient stresses occurred, i.e. when ${\mathrm{NI}}_{N}\times {\mathrm{NI}}_{P}\times {\mathrm{NI}}_{K}$ had a value of e.g. 0.8, the *NPKI* was slightly smaller with a value of 0.77.

The effect of this combined nutrient nutrition index was included in the model to represent the effect of nutrient stress on crop growth, including effects on light interception and total crop growth rate:

(12) $$\left\{\begin{array}{cc} \frac{dW}{\mathrm{dt}}=\mathrm{RUE}\times {\mathrm{PAR}}_{\mathrm{int}}\times \mathrm{TRANRF}\times \left(1-\mathrm{Dormancy}\right), & \mathrm{when}\;\mathrm{TRANRF}\leq \mathrm{NPKI}\\ \frac{\mathrm{dW}}{\mathrm{dt}}=\mathrm{RUE}\times {\mathrm{PAR}}_{\mathrm{int}}\times \mathrm{NPKI}\times \left(1-\mathrm{Dormancy}\right), & \mathrm{when}\;\mathrm{TRANRF}>\mathrm{NPKI} \end{array}\right\}$$

*PAR*_*int*_ (MJ PAR m^−2^ d^−1^) is defined as the amount of light intercepted by the crop per day and per m^2^; where *RUE* (g DM MJ^−1^ IPAR) refers to the radiation use efficiency. Growth rate is expressed as $\frac{\mathrm{dW}}{\mathrm{dt}}.$ The transpiration reduction function *TRANRF* accounts for growth rate reduction due to water limitations. The binary *Dormancy* variable switches from 0 to 1 when cassava goes into dormancy after a prolonged period of drought when soil water contents and LAI are below critical thresholds (Ezui et al., 2018), and switches from 1 to 0 when soil water contents increase above these thresholds.

### 2.8 Model parameterization and calibration

For the model parameterization, only observed data from the Edo experiment in 2016 were used. The experimental field in Edo experienced no drought as there was abundant and well distributed rainfall during the 2016 growing season (approx. 3000 mm), with measured rooting depth of over 3.2 m, ensuring sufficient soil moisture supply during the short dry season. The field was located at 7.05ºN, 6.13ºE. Soil concentrations of available P, K, Ca, and Mg were below standard critical nutrient concentrations for crop production, indicating poor soil nutrient status of the experimental sites (Adiele et al., 2020). The experiment contained three blocks, with 12 fertilizer treatments randomized within these blocks and plot size was 10 m by 8 m where fertilizer applications rates were 0, 150, and 300 kg N ha^−1^, 0, 40 and 100 kg P ha^−1^ and 0, 60, 120, 180, 240 and 300 kg K ha^−1^ in various combinations (Table S8). The widely cultivated cassava variety TME 419 was planted on May 24, 2016 at the onset of rainfall. Cassava was harvested about 4, 8 and 14 months after planting (MAP) from a net plot of 6.4 m^2^ (eight plants) of each experimental plot. At each harvest, storage roots, leaves and stem weights were measured. Sub-samples of about 400 g fresh weight were collected in the field using a digital field scale, and oven dried at 60 °C until constant weight and weighed to allow dry matter (DM) yield to be calculated. Dried subsamples from leaves with petioles, stems, and storage roots were analysed for total N, P and K concentration, using standard protocols of the isotope laboratory of the Division of Soil and Water Management, KU Leuven, Belgium. Total N in the tissue was analysed by Dumas combustion using a Carlo Erba EA1108 elemental analyser. Total P and K concentrations were measured with inductively coupled plasma (ICP) (iCAP 7400, Thermo Fisher Scientific, USA).

Parameters related to water limited crop production such as specific leaf area (*SLA*), light extinction coefficient (*k*), radiation use efficiency (*LUE_OPT*) and soil hydraulic properties at water content saturation (*SAT*), air dry (*AD*), field capacity (*FC*) and wilting point (*WP*) were derived and elaborately explained by Adiele et al. (2021b). Other required model parameters and values are in Table S7. Weather data were obtained from the nearest weather station located approximately 61 km from the field at (6.33°N, 5.60°E).

The measured biomass of leaves, stems and storage organs for intermediate and final harvests and N, P and K uptakes in plant parts were obtained from Adiele et al. (2021a), and can be found in Table 3 and in the supplementary information (Tables S1, S3, S4 and S5). The model requires input on the amounts of N, P and K that a soil can supply. As N, P or K uptakes strongly increase when other nutrients are applied, measurements or estimates of potential extra nutrient supply needs to be provided. Therefore, N, P and K uptakes derived from the control and extra N uptake from PK, extra P from NK and extra K from NP treatment need to be provided. If these uptake values for N, P and K are unknown, they can be estimated from yields in an unfertilized field, assuming that nutrient harvest indices and concentrations in storage roots are reasonably balanced. In this study, these values were derived from measurements. Parameters for nutrient limited crop growth are outlined in Table 1. Where possible, parameters were derived from measured values, including those for the maximum recovery efficiency for applied fertilizers. For some parameters values were set based on expert knowledge. Three unknown parameters were calibrated (*K*_*NI*_*, KMAX*, and *TSUM_NPKI*) to best match measured N, P and K uptakes and dry matter accumulation in plant organs at 4, 8 and 14 months for all treatments in Edo, 2016 (Table S8). The optimization sought to minimize the root mean square error (RMSE) between the measured and simulated values of DM storage root yield, N, P and K uptakes.

### 2.9 Model testing

For the model testing, fully independent data from three other field experiments from 2017 were used. The three experimental fields were located in three agro-ecological zones (Rainforest – Cross River, Rainforest Transition - Edo, and Guinea Savanna - Benue), where most of the cassava is grown in Nigeria. The fields were located at (6.80ºN, 6.23ºE), (5.96ºN, 8.77ºE) and (7.27ºN, 8.19ºE) respectively for Edo, Cross River and Benue. These experiments had the same design, planting density and data collection schemes as the experiment in Edo 2016 (used for model parameterisation and calibration). Weather data were obtained from the closest weather stations at each location in 2017, at approximately 6.76ºN, 8.69ºE in Benue and 5.97ºN, 8.72ºE in Cross River, while for the field in Edo weather data where derived from (6.33°N, 5.60°E). In order to assess the reliability of the model in diverse environmental conditions and soil N, P and K supply, measured and simulated results from the three different experiment fields were compared using the root mean square error of prediction (RMSEP) to assess model error, the coefficient of determination (*R*^*2*^) and slope of a linear relationship between measured and simulated values to assess repeatability and bias. To this end, these accuracy indicators were firstly determined for treatments that were not used to estimate soil N, P and K supply (excluding control, N0PfKf, NfP0Kf and NfPfK0 treatments) and secondly for all treatments.

### 2.10 Statistical analysis

Treatment effects on biomass components (leaves, stem and storage roots) were analysed separately for each growth stage, using a linear mixed model with biomass components as response variable and fertilizer treatment as explanatory factor, while either year or location were considered random effects. The interaction of nutrient uptake response with location and year or treatment were analysed with a mixed linear regression model. Effects were analysed with a type-III ANOVA using Satterthwaite’s approximation method. Differences between treatment means were considered significant when probability ≤ 0.05. R software (R Core, 2019), version 3.5 with the lme4, lmerTest, and Predictmeans packages was used for statistical analysis.

## 3 Results

### 3.1 Observed nutrient uptake

Nutrient uptake by the plant components increased with nutrient availability. Leaf N uptake differed by treatment and location. There was no interaction between treatment and location (Table S2). Leaf N uptake was largest in the NfPfKf treatment with an average of 11 g m^−2^, between Edo and Cross River where locations did not differ significantly. Leaf P and K uptake differed by treatment and location, while the K uptake differed only by treatment. The largest leaf P uptake in 2016 was recorded in Edo, with the largest leaf K uptake in Cross River. Stem N uptake was largest in the NfPfK180 with an average of 18. 0 g m^−2^, while the stem P uptake was largest in the NfPfKf treatment, with 2.0 g m^−2^ (Table 3). Interaction between treatment and location for stem K uptake was significant (p > 0.001), with uptake increasing with application rates as expected (Table S2). Uptake of N and P by the storage roots was significantly different between treatments and locations (p > 0.001). Storage root K uptake was only significantly different among treatments (p > 0.001), with the greatest uptake in the NfPfKf treatment (Table 3).

### 3.2 Dry matter yield of measured plant components (leaves, stems and storage roots)

Leaf DM at about 4 MAP differed between location and treatment (Tables S3 and S4), while at 8 MAP, leaf DM differed only among locations in both years Tables S5 and S6. At 4 MAP, year average leaf DM ranged from 36.9 g m^−2^ in the control treatment at Benue to 350 g m^−2^ in the NfPfKf treatment at Edo. Leaf DM decreased across all treatments, years and locations at 8 MAP (seasonal dry period). At final harvest, leaf DM differed by treatment and location (Tables S7 and S8) and was highest in the NfPfKf treatment (319 g m^−2^) from Edo. Interaction between treatment and location was significant for stems DM at 4 MAP and final harvest (p < 0.01) (Table S6). At 4 MAP, year average stem DM ranged between 55 g m^−2^ in the control treatment at Benue and 492 g m^−2^ in the NfPfKf treatment at Edo. The interaction between treatment and location for storage roots DM was significant (p < 0.01) at 4 MAP (Table S6). The amount of storage roots was lowest at Benue during this stage. Storage root DM at final harvest differed by treatment and location (p < 0.001) (Table S6), ranging from 588 g m^−2^ in the control treatment at Benue to 3547 g m^−2^ in the NfPfKf treatment at Edo. Overall plant biomass decreased with decreasing fertilizer rates.

### 3.3 Model calibration results

**Fig. 1:**
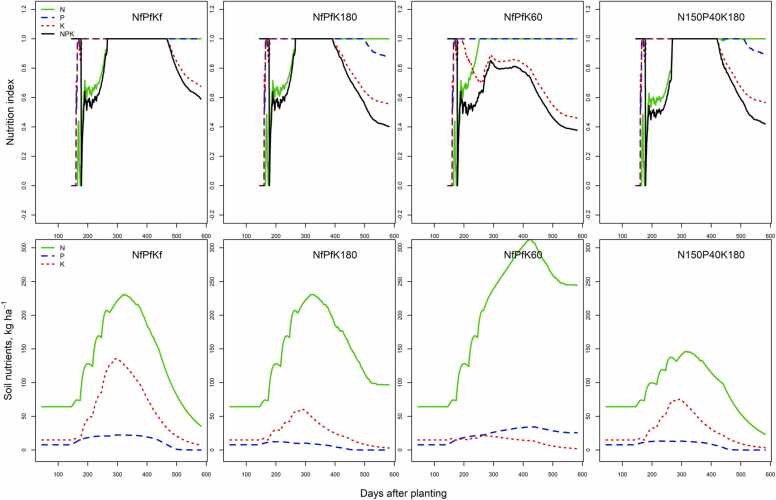
Results of simulated N, P and K nutrition indices, and relative interaction among N, P and K nutrition indices (NPK) and soil nutrients available (kg ha^−1^) of treatments used for calibration from Edo in 2016.

**Fig. 2:**
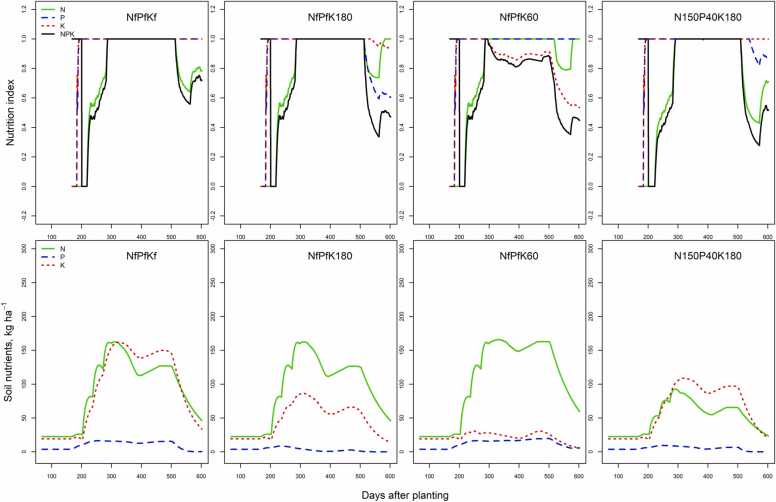
Results of simulated N, P and K nutrition indices, and relative interaction among N, P and K nutrition indices (NPK) and soil nutrients (kg ha^−1^) of treatments used for model testing from Cross River in 2016.

After calibration, the N, P, K uptakes and yields simulated by the model were close to the 2016 measurements in Edo with a difference between the observed and simulated stems and storage root yields at final harvest of 15% and 23%, respectively (Fig. S5). However, green leaf DM was underestimated by the model. Nevertheless, LINTUL-Cassava-NPK simulated total leaf weight (sum of green and dead leaves) reasonably well, when petioles were assumed to be 20% of the total leaf weight (Adiele et al., 2021b). Simulated N, P and K nutrition indices responded to the various treatments (Fig. 1). All initial *NI*_*N*_ values were below optimum, with the lowest index value for the 150 kg N ha^−1^ treatment. The treatments with 180 kg K ha^−1^ were K deficient and reduced simulated crop growth rates towards the end of the growing season. In the NfPfK60 treatment, P supply was sufficient for optimum crop growth throughout the season, while K was deficient and restricted simulated crop growth rates from day 190 onwards. The calibrated model simulated a storage root yield of 1332 g m^−2^, below the observed yield of 1969 g m^−2^. In the NfPfKf treatment, N and P supply were adequate to support growth, but K supply was suboptimal (NI_K_ = 0.7) from about 70 days to the end of the growth season. The simulated soil nutrient availability during the growing season showed that the NfPfK60 treatment had a surplus of N in the soil at the end of the growing season (Fig. 1). The calibrated model simulated well the N, P and K nutrition indices and soil nutrient in Cross River 2016 (Fig. 2). Similarly, N supply was limited at the beginning of the season. Potassium supply was adequate for crop growth in the NfPfKf and N150P40K180 treatments. Shortage of N (NI_N_ = 0.7) affected growth in the NfPfKf treatment from about 69 days to the final harvest, while in the N150P40K180 treatment, N and P were insufficient from 82 and 28 days to the final harvest, respectively (Fig. 2).

**Fig. 3:**
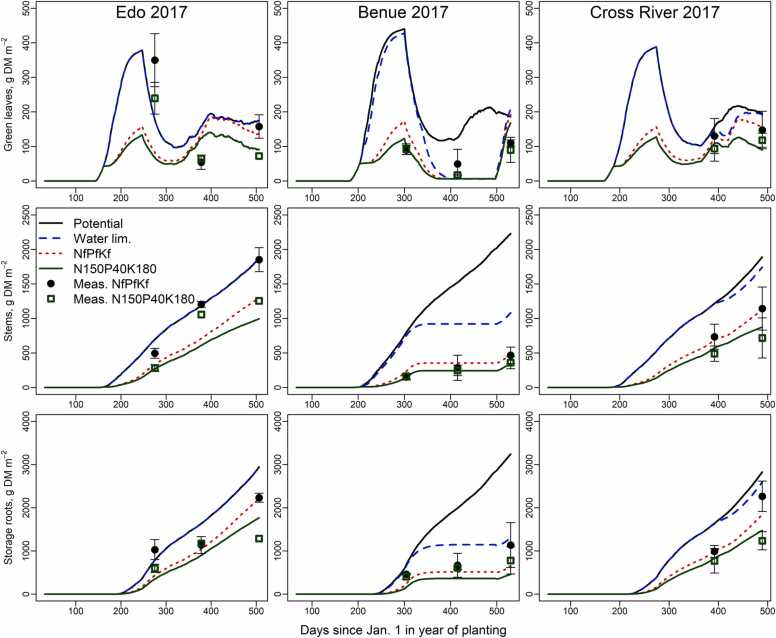
Observed and simulated green leaves, stems and storage root yield DM (g m^−2^) under water limited (Water lim.), and nutrient limited conditions (NfPfKf and N150P40K180 treatments) from Edo, Benue and Cross River in 2017. Measured (Meas.) values are shown with error bars indicating one standard deviation. Observations for the first harvest at 4 months after planting were not available for Cross River.

Simulated nutrient limited growth of green leaves, stem and storage root DM for the optimized (NfPfKf) treatment showed a good match with observed values, in the test with other locations in 2017 (Fig. 3). Most especially, the interaction between soil moisture and nutrient availability for crop use and its effect on yield was well captured at Benue 2017, where shortage of soil moisture hampered nutrient uptake, and subsequently cassava growth (Fig. 3). All organs of the N150P40K180 treatment had lower simulated values than the NfPfKf treatment for Edo and Cross River (Fig. 3), explaining nutrient limitations for this treatment..

### 3.4 Model testing results

**Fig. 4:**
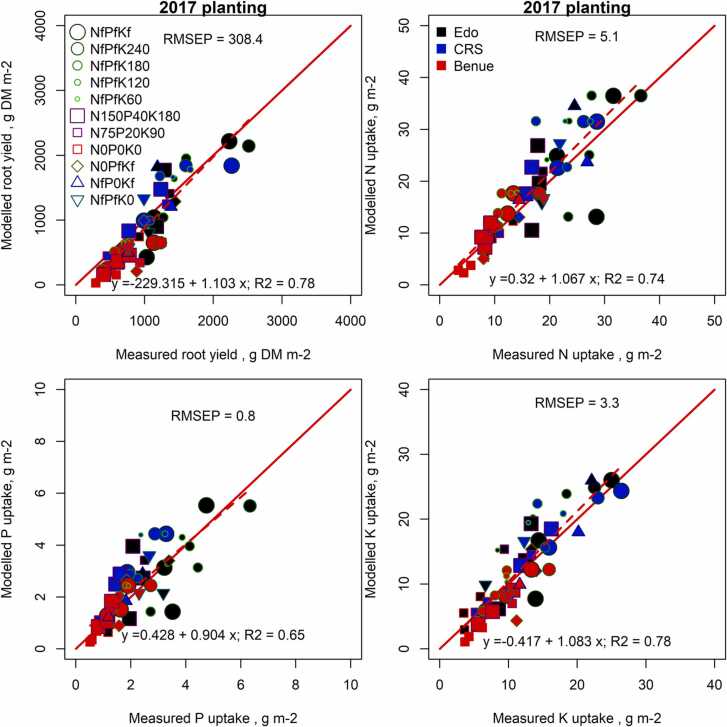
Observed and simulated storage roots, N, P and K uptakes of both non-nutrient and nutrient limiting conditions from Edo, Cross River (CRS) and Benue in 2017 at 4 and 8 month after planting and at final harvest. The solid line is the 1:1 line, while the dashed line is the best fit regression.

The simulated values of storage root yield, N, P and K uptakes were in good agreement with the observed uptakes for the selected treatments (Fig. 4), excluding treatments used to estimate soil N, P and K supply, which are the control, N0PfKf, NfP0Kf and NfPfK0 treatments (Fig. S7). The RMSEP obtained, indicating the mean of the absolute difference of observed and simulated values for all treatments, was 308 g storage root DM m^−2^ with a *R*^*2*^ of 0.8 for the linear relationship between measured (observed) vs. modelled (simulated) yields. The simulation of total N, P and K uptake resulted in a RMSEP and a R^2^ value of 5.1 g m^−2^ and 0.7 for N, 0.8 g m^−2^ and 0.7 for P and 3.3 g m^−2^ with R^2^ of 0.8 for K. The observed data are also shown in Table 3 and S1 for uptakes and Table S5 for storage root dry matter yield.

## 4 Discussion

### 4.1 Nutrient uptake and yield

Cassava accumulated more nutrients when more were supplied, as expected. The largest sinks for the nutrients were storage roots, followed by stems and leaves (Table 3 and S1). Such a nutrient uptake pattern was expected since there was more DM in storage roots (Ichinose et al., 2018). The differences in nutrient uptake among the treatments clearly show that crop growth was limited by the inadequate nutrient supply due to poor soil fertility. Besides limiting yields, inadequate nutrient supply further restricts the nutritional composition of crops, thereby altering their nutritional quality (Imakumbili et al., 2019). Further, soil water availability influenced nutrient uptake as observed through differences among locations, with cassava accumulating more nutrients throughout the season at Edo where little effect of drought was observed. Leaf weight did not differ among treatments at 8 MAP. Rather, it generally decreased across locations and treatments in the middle of the cropping season (8 MAP) due to assimilate partitioning in synchrony with the mid-season drought. Stem and storage root yield increased with fertilizer application. The harvest index of dry matter was smaller for treatments without P or K fertilization (Fig. S1), and increased proportionally with K supply (Fig. S2). Phosphorus encourages formation of storage roots in cassava (Pellet and El-Sharkawy, 1993). Although this process was not included in the model, simulated proportions of biomass in leaves, stems and storage organs varies with nutrient supply resulting from cassava growth rate reductions at various stages of development. Also leaf biomass increased early in the season with fertilizer application, resulting in adequate LAI in the dry season with crops that were less affected by drought effects at 8 MAP, especially at Edo where the deep-rooted crop had access to deeper soil water reserves (Adiele et al., 2021b). Nutrient demand in this drier period was however strongly reduced as dying leaves provided enough nutrients (Adiele et al., 2021a). Further, with some partial stomatal closure during dry periods with a large vapor pressure deficit (El-Sharkawy et al., 1992; Raji and Byju, 2020), cassava continues to accumulate dry matter in its storage roots, though at a reduced rate. After the dry period, nutrient uptake and yield increased in all treatments and locations, but was larger in optimized fertilizer treatments than in the control and treatments with lower fertilizer rates, suggesting that soil available N, P and K pools were larger as well.

### 4.2 Model performance

LINTUL-Cassava-NPK combines the essential crop growth processes described in LINTUL–Cassava (Ezui et al., 2018) with the major plant and soil N, P and K processes. The low *NI*_*N*_ indicates insufficient N supply in the first part of the season before fertilizer application in all experiments. Thereafter, N supply was adequate in Edo throughout the growth period, including the treatment with 150 kg N ha^−1^. This could be related to the relatively fertile soils compared with the other locations and the absence of leaching due to deep rooting of cassava at Edo (Adiele et al., 2020a). In the NfPfK60 treatment, P supply was sufficient to support crop growth without stress throughout the growing season, as was N supply after fertilizer application (Fig. 1). However, modelled K supply was sub-optimal from 34 days after the initial split fertilizer application of 20 kg K ha^−1^ onwards, resulting in a moderate but consistent reduction of water limited potential growth. In this study, a large N rate (300 kg N ha^−1^) with increasing rates of K fertilizer was studied. At low K supply, yield response to the large N rate was strongly depressed. Seasonal trends of the nutrition indices and soil nutrient availability during the growing season in Cross River 2016 were simulated reasonably well by the model (Fig. 2), also indicating insufficient N supply at the beginning of the season. Potassium supply was sufficient for crop growth in the NfPfKf and N150P40K180 treatments, but growth was later suppressed by insufficient N supply in the NfPfKf, while shortages of N and P supply contributed more to growth reduction in the N150P40K180 treatment (Fig. 2). Imbalanced nutrient combinations, for instance in the NfPfK60 resulted in an excess of N in the soil, although not to the same extent as in Edo. This could be related to the relatively strong indigenous soil K supply in the Cross River sites when compared to the sites in Edo.

The model simulated reasonably well the dynamics of cassava organ growth and dry matter production under nutrient-limited conditions (Fig. 3). The observed storage root yield at final harvest in the NfPfKf treatment was in good agreement with the simulated nutrient-limited yield across the locations and was larger than the yield from the nutrient-limited treatment N150P40K180. Observed storage root yield in the N150P40K180 treatment at final harvest was far less than that simulated in Edo and Cross River, where growing conditions were favourable, resulting in large and significant increases in storage root yield at higher fertilizer application rates. Moreover, the model tests results show that the effect of nutrient stress on dry matter production is well simulated. The prediction accuracy of storage root yield, N, P and K uptakes was satisfactory (Fig. 4) with linear relationships between simulated and observed values, a slope close to 1, good repeatability (*R*^*2*^ of 0.7 – 0.8) and only a minor bias. The modelling approach used is relatively simple and uses a single value for RUE and fixed values for proportional growth of organs as function of development stage. The stomata of cassava respond to vapour pressure deficits (VPD) (Vongcharoen et al., 2018), reducing net photosynthetic rates and radiation use efficiency. In our experiments, high VPD values were only present in the dry season when plants were already drought stressed. In Edo, cassava did not suffer from drought in 2016 and maintained a high RUE in the dry season despite relatively high VPD, suggesting that VPD did not have a strong influence. These observations suggest that effects of VPD can be considered as one component of the response to drought for the climates as observed in southern Nigeria. In LINTUL-Cassava-NPK this is captured with a transpiration constant that, in combination with potential transpiration rates, determines the critical soil water content below which crops reduce growth rates (Ezui et al., 2018). Effects of nutrient availability on the proportional growth of specific organs and harvest indices were significant (see supplementary materials) but estimated effects were small and were purposely ignored as this process requires detail knowledge about redistribution, with little influence on modelled results.

### 4.3 The nutrient equivalents modelling approach

Measured crop N, P and K uptakes were more or less proportional to soil available nutrients, as if the crop was aware of total amounts present in the soil, with e.g. lower N uptake if K is limiting or vice versa. To balance the nutrient uptake with soil supply, both demand and supply were expressed as nutrient equivalents. Both total nutrient equivalent uptake and actual uptake of an individual nutrient were limited by soil supply, ensuring that proportionally more N and P were taken up when K was limited, while much less N and P were taken up than would be possible with a perfectly balanced N, P and K supply in the soil. The idea of nutrient equivalents (Janssen, 2011) was developed with the model QUEFTS in mind. When the three major nutrients, N, P and K, are expressed in terms of nutrient equivalents, it becomes possible and reasonable to calculate the fraction of each nutrient taken up in the sum of the three (Janssen, 2011). Results in our experiments show that each nutrient was taken up in proportion to its supply (Adiele et al., 2021a). The nutrient equivalent approach that was adopted in the model ensures that nutrients are not excessively accumulating in leaves or other organs when other nutrients are limiting. As a result, leaf growth is strongly limited by nutrient deficiency and is maintained within a narrow range of nutrient concentrations. It also ensures that nutrients do not accumulate easily in other organs when the soil nutrient supply is unbalanced. The nutrient equivalent approach to determine N, P and K uptake, differs from the approach used in the SIMCAS model which does not consider the interaction between uptake of N and K (Mithra et al., 2013). In our approach, the actual uptake determined the nutrition indices for N, P and K, comparing actual uptake with minimum and optimum uptake following the nitrogen nutrition index approach (Greenwood et al., 1986, Shibu et al., 2010).

Furthermore, plants do not only strive for an optimal ratio of nutrients in their tissues but also need to remain neutral with a balance between cations and anions, a condition necessary for maintenance of good growth (De Wit et al., 1963, Hawkesford et al., 2012). As long as nitrate is not exhausted, anions are taken up in excess to the cations. During this process of excess anion uptake, electroneutrality is maintained by a net uptake of H^+^ ions. As growth continues, the nitrate is eventually exhausted and from then on cations are taken up in excess of anions (De Wit et al., 1963). This was also observed in our study (Adiele et al., 2021a), where more N than K was taken up at early growth stages. The nutrient equivalents approach adopted here ensures a reasonable balance between uptake of nitrate and orthophosphate anions and K cations. The strong and linear response to K observed can be understood when considering the large N supply and uptake of cassava, where K is the dominant cation to counterbalance anions in the cytoplasm (Hawkesford et al., 2012) and is needed to extrude excess anions (De Wit et al., 1963) enabling uptake of the nitrate anion.

Under imbalanced supply of nutrients, plants take up more of the omitted nutrients (Pasley et al., 2019) due to improved soil exploration by more developed root systems and/or root functioning. This plant-soil interaction is not modelled, we used input values for indigenous nutrient supply and fertilizer recovery derived from experimental measurements (Table 2) as parameters in the LINTUL-Cassava-NPK model. However, we hypothesise that the proportional increase in soil supply of an omitted nutrient depends on the amounts of the other nutrients applied. Also, partitioning of the total N, P and K uptake over the leaves, stem and storage roots was based solely on the proportional weight of each organ. Though partitioning is not explicitly modelled, it does not affect overall nutrient uptakes and yield responses. To better model this aspect, nutrient allocation in the plant and organ specific limitations for N, P and K need to be described. We do not yet have the data to test such approaches. Moreover, we do not consider it realistic to attempt to simulate a complete description of plant-soil nutrient interactions influencing cassava growth and yield under nutrient-limited conditions given the lack of detailed information on nutrient availability. The model is based on the Monteith approach with one RUE parameter that reflects the average RUE for the whole growing season when water and nutrients are not limiting. Further, the partitioning of assimilates is a function solely of crop development. In our model, nutrient limitations have a direct effect on crop growth rates. The reduction in growth rates depends on the soil supply but also on the demand of the growing organs. Nutrient deficiency therefore reduces growth rates strongly when leaves are growing with a high demand, resulting in an adjustment of LAI and consequently light interception. Secondly, effects of nutrient deficiency on simulated leaves, stem and storage organ biomass differed, aligning with differences in nutrient demand and supply when organs are growing. The model captured the characteristic response of cassava to nutrient deficiency with a strong effect on LAI (and canopy size) and proportions of biomass in the various organs and harvest index, without adjustment of parameters for biomass partitioning. Also proportions of N, P and K uptake in the storage roots differed among treatments (Fig. S1). We expect that the model concepts are fairly robust and can be used in conditions that are similar to those in southern Nigeria, with planting 3–5 months before a dry period with reduced plant activity, followed by a sufficiently wet period to harvest. We recommend to test if parameter values are adequate for situations that strongly differ, e.g. when planting outside the typical planting window or when simulating for very different climates. Our approach illustrates, for the first time, how the interactions of N, P and K limitations impact cassava growth rates and storage root yield under rainfed conditions, using a dynamic simulation model.

**Table 2:** Uptake and recovery ± standard error of N, P and K from soil (kg ha^−1^) as measured in control plots with additional uptake for Benue, Cross River (CRS) and Edo in the 2016 and 2017 growing periods.

Site	Uptake in control treatment			Additional uptake in omission treatments^a^		
	N, kg ha^−1^	P, kg ha^−1^	K, kg ha^−1^	N, kg ha^−1^	P, kg ha^−1^	K, kg ha^−1^
	Calibration					
Edo, 2016	194.2 ± 81.5	19.9 ± 6.7	88.0 ± 25.7	62.7 ± 105.3	11.5 ± 12.4	-27.9 ± 26.1
	Testing					
Benue, 2017	56.5 ± 13.3	7.6 ± 2.6	61.9 ± 30.6	23.1 ± 24.1	4.3 ± 4.5	38.0 ± 96.6
CRS, 2017	108.5 ± 46.7	11.1 ± 7.0	69.0 ± 48.1	35.8 ± 40.1	7.1 ± 12.1	53.8 ± 75.2
Edo, 2017	133.6 ± 50.1	15.6 ± 3.9	59.2 ± 34.6	47.1 ± 21.2	47.1 ± 21.2	7.2 ± 23.1

a Additional N, P and K uptake as measured in the treatments with 0:100:300, 300:0:300 and 300:100:0 kg N:P:K ha^−1^, respectively

**Table 3:** Predicted means and confidence interval (0.05) for factors included in a mixed effect model explaining uptake of N, P, and K in leaves, stem, and storage roots for cassava. Nutrient application rates per treatment, (f) represents full rate of the optimized nutrient of N, P and K at 300, 100 and 300 kg ha^−1^ with year as random factor.

Site and treament	Leaves	Conf. interval		Stem	Conf. interval		Roots	Conf. interval	
	Pred. means	Lower	Upper	Pred. means	Lower	Upper	Pred. means	Lower	Upper
			N uptake, g m^−2^						
**Benue**									
Control	2.34	-1.60	6.29	1.37	-2.78	5.52	2.28	-0.41	4.98
NfPfK180	4.17	0.22	8.12	3.65	-0.50	7.79	5.34	2.64	8.03
NfPfKf	5.24	1.30	9.19	5.19	1.04	9.34	8.59	5.89	11.28
N150P40K180	3.98	0.04	7.93	2.36	-1.78	6.51	4.97	2.28	7.67
**Cross River**									
Control	3.61	-0.34	7.56	2.60	-1.54	6.75	4.42	1.72	7.11
NfPfK180	8.55	4.61	12.50	7.97	3.82	12.12	7.89	5.20	10.57
NfPfKf	9.66	5.71	13.60	9.97	5.83	14.12	13.01	10.32	15.70
N150P40K180	5.36	1.42	9.31	5.94	1.80	10.09	7.30	4.61	10.00
**Edo**									
Control	6.26	2.31	10.20	5.33	1.19	9.48	4.79	2.10	7.49
NfPfK180	7.71	3.76	11.65	17.96	13.81	22.11	11.72	9.03	14.42
NfPfKf	11.07	7.12	15.01	14.26	10.11	18.41	16.31	13.62	19.00
N150P40K180	5.59	1.64	9.53	10.10	5.95	14.25	8.94	6.25	11.64
									
			P uptake, g m^−2^						
**Benue**									
Control	0.15	-0.13	0.43	0.16	-0.30	0.61	0.45	-0.002	0.91
NfPfK180	0.25	-0.03	0.53	0.42	-0.03	0.88	1.07	0.61	1.52
NfPfKf	0.34	0.06	0.62	0.62	0.16	1.07	1.65	1.19	2.10
N150P40K180	0.26	-0.02	0.54	0.27	-0.18	0.72	0.89	0.43	1.34
**Cross River**									
Control	0.22	-0.06	0.50	0.29	-0.17	0.74	0.69	0.23	1.14
NfPfK180	0.51	0.23	0.79	0.83	0.37	1.28	1.23	0.77	1.68
NfPfKf	0.69	0.41	0.96	1.17	0.71	1.62	1.95	1.50	2.41
N150P40K180	0.31	0.03	0.59	0.52	0.07	0.97	1.03	0.57	1.48
**Edo**									
Control	0.44	0.16	0.72	0.59	0.13	1.04	0.75	0.29	1.20
NfPfK180	0.60	0.31	0.88	2.31	1.85	2.76	1.70	1.24	2.15
NfPfKf	0.92	0.64	1.20	2.00	1.54	2.45	2.70	2.25	3.16
N150P40K180	0.42	0.14	0.70	1.05	0.60	1.51	1.20	0.74	1.65
									
			K uptake, g m^−2^						
**Benue**									
Control	0.71	-0.32	1.75	1.31	-0.04	2.66	5.03	0.68	9.37
NfPfK180	1.38	0.35	2.42	3.75	2.40	5.10	9.95	5.61	14.30
NfPfKf	1.91	0.88	2.95	4.78	3.43	6.13	15.97	11.62	20.3
N150P40K180	1.35	0.32	2.39	2.77	1.42	4.12	9.27	4.92	13.62
**Cross River**									
Control	0.63	-0.41	1.66	1.31	-0.04	2.66	5.61	1.26	9.95
NfPfK180	2.22	1.18	3.26	6.03	4.68	7.38	10.34	5.99	14.68
NfPfKf	2.52	1.48	3.55	7.08	5.73	8.43	16.69	12.34	21.04
N150P40K180	1.25	0.21	2.29	4.58	3.23	5.93	10.06	5.71	14.41
**Edo**									
Control	1.17	0.13	2.20	1.80	0.45	3.15	4.40	0.05	8.74
NfPfK180	1.77	0.73	2.81	8.50	7.14	9.84	8.97	4.62	13.32
NfPfKf	3.22	2.18	4.25	10.27	8.92	11.62	15.21	10.86	19.56
N150P40K180	0.92	-0.11	1.96	4.73	3.38	6.08	8.00	3.65	12.35

## 5 Conclusion

The LINTUL-Cassava-NPK model simulates the effects of stress due to inadequate N, P and K supply on nutrient uptake, crop growth rates and yield of cassava. The model was built based on observed data from field experiments, with simulated nutrient uptakes and storage root yields in good agreement with observed nutrient uptakes and storage root yields. Our model results suggest that more N is needed at the onset of the season, whereas K needs to be applied at larger amounts and at a later growth stage. This model can help to better understand spatial and seasonal variations in nutrient recovery and use efficiency, enabling the development of improved nutrient management recommendations, with respect to space, timing and quantity, to improve efficiency of applied nutrients with reduced losses and maximum storage root yields.

## Declaration of Competing Interest

The authors declare that they have no known competing financial interests or personal relationships that could have appeared to influence the work reported in this paper.
