# Supplementary material for: LINTUL-Cassava-NPK: A simulation model for nutrient-limited cassava growth
Source: Field Crops Res. 2022 May 15;281:108488. doi: 10.1016/j.fcr.2022.108488 (PMC8935378; doi:10.1016/j.fcr.2022.108488)
Supplement: Supplementary file 1 — Supplementary material. [file mmc1.docx]

Supplementary materials

**LINTUL-Cassava-NPK: a simulation model for nutrient-limited cassava growth**

**J.G. Adiele^a,b^, A.G.T. Schut^a,*^, K.S. Ezui^c^, K.E. Giller^a^**

^a^Plant Production Systems Group, Wageningen University, P.O. Box 430, 6700 AK, Wageningen, The Netherlands

^b^National Root Crops Research Institute, Umudike, KM 8 Ikot Ekpene Road, P.M.B 7006, Umuahia

Abia State, Nigeria

^c^African Plant Nutrition Institute, ICIPE Campus, Duduville – Kasarani, Thika Road, Nairobi, Kenya

*corresponding author: P.O. Box, 430, 6700 AK, Wageningen, The Netherlands.

Email address: tom.schut@wur.nl

Methods

Differences between treatments were analysed with a regression model. A linear model was fitted to the observed harvest indices for DM, N, P and K and proportions of biomass and N, P and K in the various organs. The linear model included factors for treatment and interactions between year and location (y ~ treatment + year * location). Predicted means were computed to analyse the effect of treatments on nutrient uptake at the first harvest at 4 months after planting (MAP), second harvest at 8 MAP and final harvest. Interaction effects of treatment and location at final harvest in leaves, stems and storage roots were evaluated with a mixed linear model with year as random effect. An ANOVA with F-test was used to test significance of treatment × location effects. For this, R software with the lme4, lmerTest and predictmeans packages were used.

Results

Nutrient limitations affected the harvest indices for DM, N, P and K (Fig. S1). Interactions between location and year were strongly significant (P<0.001). The HI of the 0 kg P/ha (P<0.01) and 0 kg K/ha (P<0.001) treatments were significantly lower than the control and full NPK treatments, while the 0 kg N/ha treatment was not different. However, the estimated effects are small, the harvest index was 0.075 and 0.072 lower for the 0P and 0K treatments respectively. This indicates that ignoring this process doesn’t strongly affect the predicted yields. To better model this aspect, also nutrient allocation in the plant and organ specific limitations for N, P and K need to be described. Our results suggest that P limitation is a key determinant for N and K allocation to roots, evidenced by the lower harvest index values for N, P and K for the P limited treatment (Fig. S1). Limitations of N or K did not strongly affect proportional allocation of N and K to storage organs. P allocation to storage organs was affected by K supply, resulting in lower P harvest index values for the treatments without N and K application. Treatments with varying amounts of K (Fig. S2), treatments varying in amounts of N, P and K supply (Fig. S3) and N, P or K omission treatments with control and the full N, P and K application treatment (Fig. S4), did not differ in the proportional distribution of biomass over leaves, stems and storage organs. However, some effects of N, P or K limitation on partitioning were observed.

Tables S1, S3, S4 and S5 contain the N, P and K uptake in leaves, stem, and storage roots at final harvest and measured biomass of leaves, stems and storage organs for intermediate and final harvests. The nutrients uptake in leaves and storage roots differed by treatments and location, while the interaction between location and year were significant for P and K uptake in stems (Table S2). Also, leaves, stem and storage root biomass differed by treatment and location. However, there was interaction between treatment and location for stems at 4 MAP and final harvest, including storage roots at 4 MAP (P<0.01) (Table S6). Overall plant biomass increased with increasing fertilizer rates.


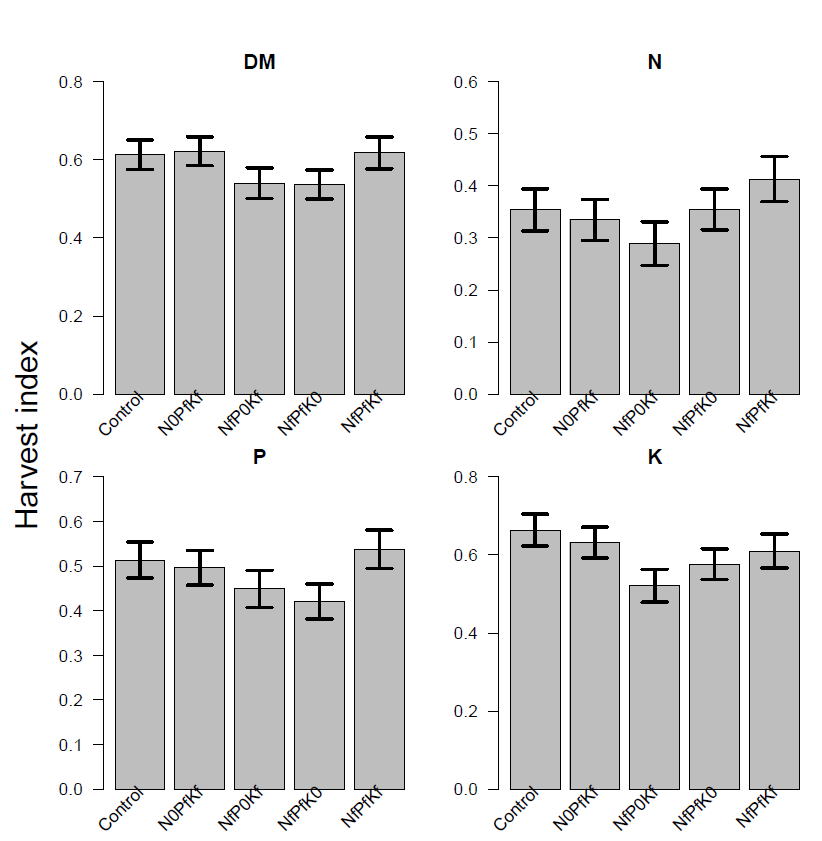


Fig S1. Predicted means for measured harvest indices for DM, N, P and K after accounting for year and location effects. Error bars indicate the 95% confidence intervals.


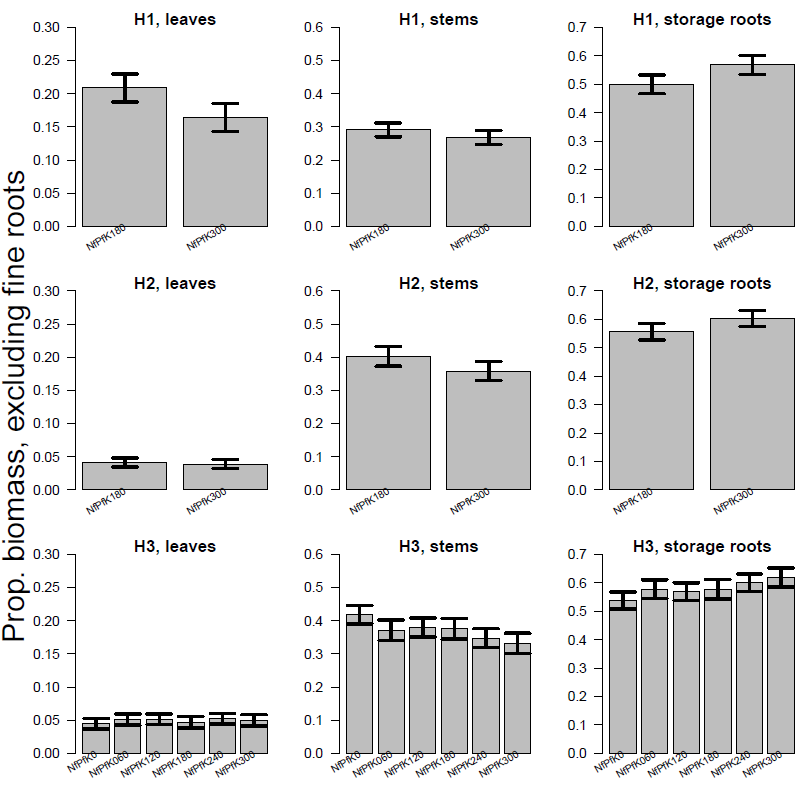


Fig S2 Predicted mean proportions of biomass in leaves, stems and storage organs at first (H1), second (H2) or final harvests (H3) after accounting for year and location effects for treatments with varying amounts of K. Error bars indicate the 95% confidence interval of the mean.


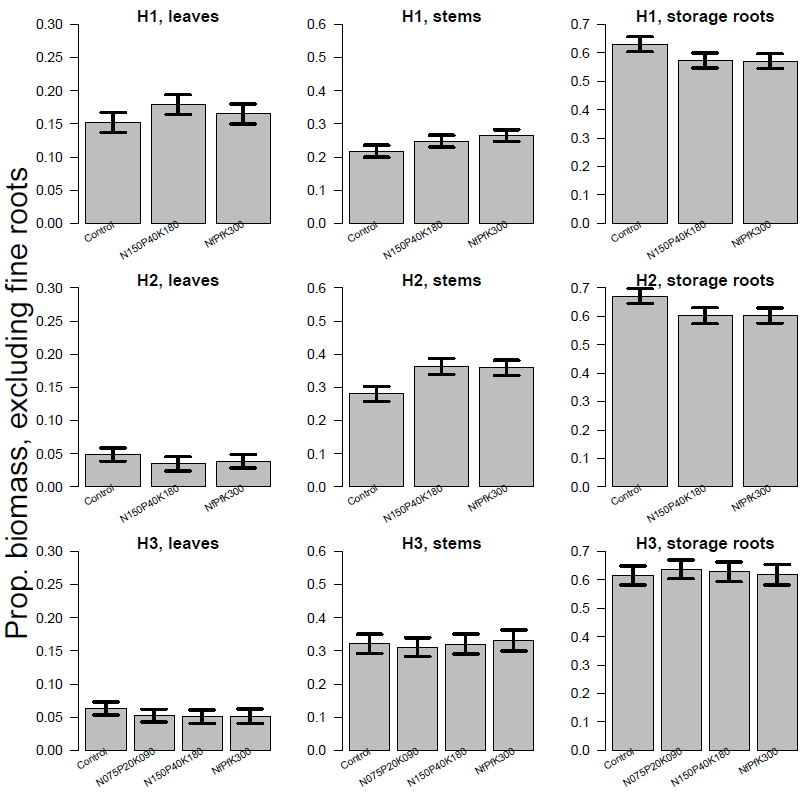


Fig S3 Predicted mean proportions of biomass in leaves, stems and storage organs at first (H1), second (H2) or final harvests (H3) after accounting for year and location effects for treatments varying in amounts of N, P and K. Error bars indicate the 95% confidence interval of the mean.


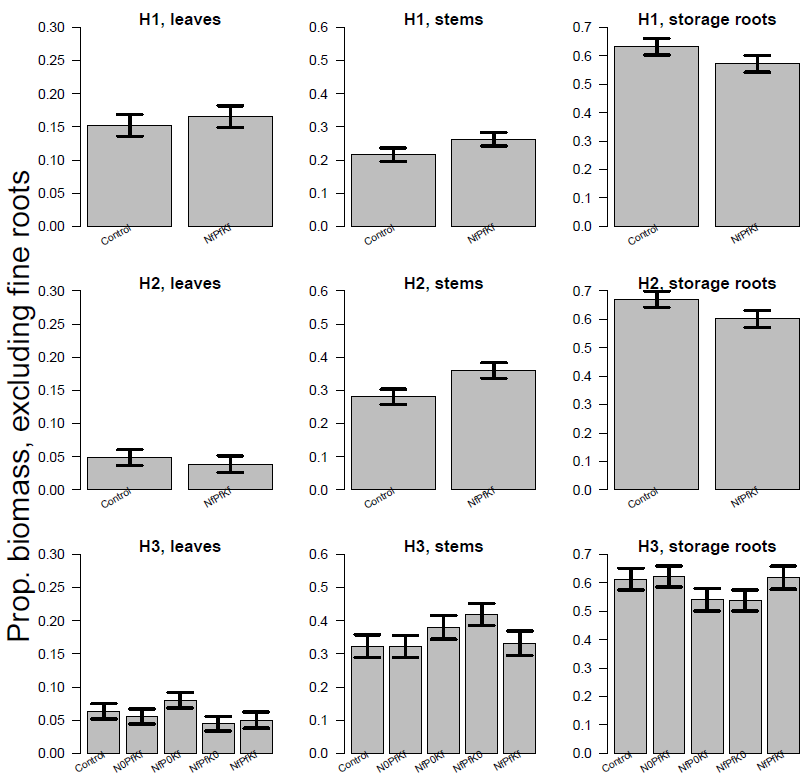


Fig S4 Predicted mean proportions of biomass in leaves, stems and storage organs at first (H1), second (H2) or final harvests (H3) after accounting for year and location effects for N, P or K omission treatments with control and the full N, P and K application treatment. Error bars indicate the 95% confidence interval of the mean.

Table S1. Uptake of N, P, and K in leaves, stem, and storage roots at final harvest for cassava in the Benue, Cross River (CRS) and Edo in the 2016-2017 and 2017-2018 growing seasons.

| Year | Treatment | Benue | | |  | CRS | | |  | Edo | | |
| --- | --- | --- | --- | --- | --- | --- | --- | --- | --- | --- | --- | --- |
|  |  | Leaf | Stem | Roots |  | Leaf | Stem | Roots |  | Leaf | Stem | Roots |
|  |  | N, g m^-2^ | | | | | | | | | | |
| 2016 | N0PfKf | 3.18 | 1.58 | 3.23 |  | 3.53 | 3.26 | 2.21 |  | 9.50 | 10.59 | 5.60 |
| 2016 | NfP0Kf | 5.61 | 4.55 | 4.09 |  | 11.01 | 8.83 | 6.22 |  | 15.07 | 15.54 | 7.99 |
| 2016 | NfPfK0 | 5.46 | 4.51 | 5.89 |  | 4.74 | 7.68 | 6.32 |  | 3.44 | 11.71 | 11.61 |
| 2016 | NfPfK240 | 5.63 | 4.34 | 4.84 |  | 8.47 | 10.13 | 9.52 |  | 13.54 | 21.32 | 10.76 |
| 2016 | NfPfK120 | 6.70 | 4.52 | 5.93 |  | 6.95 | 10.61 | 6.76 |  | 8.44 | 17.83 | 11.93 |
| 2016 | NfPfK60 | 5.38 | 3.13 | 5.28 |  | 3.77 | 6.76 | 4.56 |  | 8.49 | 22.43 | 11.30 |
| 2016 | N75P20K90 | 4.06 | 2.13 | 3.20 |  | 2.84 | 3.51 | 3.70 |  | 6.50 | 11.31 | 7.49 |
|  |  | P, g m^-2^ | | | | | | | | | | |
| 2016 | N0PfKf | 0.21 | 0.22 | 0.61 |  | 0.23 | 0.55 | 0.69 |  | 0.77 | 1.56 | 1.06 |
| 2016 | NfP0Kf | 0.28 | 0.20 | 0.48 |  | 0.47 | 0.42 | 0.63 |  | 0.97 | 1.20 | 0.97 |
| 2016 | NfPfK0 | 0.26 | 0.48 | 1.07 |  | 0.38 | 1.06 | 0.86 |  | 0.33 | 1.63 | 0.97 |
| 2016 | NfPfK240 | 0.34 | 0.43 | 0.88 |  | 0.55 | 1.14 | 1.53 |  | 0.79 | 2.75 | 1.47 |
| 2016 | NfPfK120 | 0.39 | 0.42 | 1.08 |  | 0.48 | 1.19 | 0.96 |  | 0.68 | 1.87 | 1.45 |
| 2016 | NfPfK60 | 0.32 | 0.41 | 0.98 |  | 0.27 | 0.83 | 0.78 |  | 0.74 | 3.47 | 1.54 |
| 2016 | N75P20K90 | 0.26 | 0.22 | 0.62 |  | 0.16 | 0.35 | 0.66 |  | 0.54 | 1.19 | 0.90 |
|  |  | K, g m^-2^ | | | | | | | | | | |
| 2016 | N0PfKf | 1.10 | 1.62 | 8.34 |  | 0.97 | 3.08 | 6.92 |  | 0.97 | 3.08 | 6.92 |
| 2016 | NfP0Kf | 2.08 | 2.29 | 7.90 |  | 3.45 | 6.09 | 9.71 |  | 3.45 | 6.09 | 9.71 |
| 2016 | NfPfK0 | 1.55 | 3.11 | 11.98 |  | 0.73 | 1.91 | 5.03 |  | 0.73 | 1.91 | 5.03 |
| 2016 | NfPfK240 | 1.76 | 3.79 | 11.43 |  | 2.06 | 6.60 | 12.63 |  | 2.06 | 6.60 | 12.63 |
| 2016 | NfPfK120 | 1.70 | 2.85 | 12.66 |  | 1.56 | 4.16 | 6.44 |  | 1.56 | 4.16 | 6.44 |
| 2016 | NfPfK60 | 1.59 | 2.32 | 10.61 |  | 0.74 | 3.35 | 5.79 |  | 0.74 | 3.35 | 5.79 |
| 2016 | N75P20K90 | 1.38 | 1.68 | 8.47 |  | 0.59 | 2.37 | 6.82 |  | 0.59 | 2.37 | 6.82 |
|  |  | N, g m^-2^ | | | | | | | | | | |
| 2017 | N0PfKf | 3.29 | 1.87 | 2.81 |  | 4.27 | 4.57 | 5.59 |  | 4.26 | 6.69 | 7.12 |
| 2017 | NfP0Kf | 4.00 | 4.73 | 5.70 |  | 11.33 | 7.53 | 7.96 |  | 5.37 | 12.79 | 6.35 |
| 2017 | NfPfK0 | 2.55 | 11.41 | 4.93 |  | 4.95 | 10.12 | 6.75 |  | 3.50 | 7.25 | 7.82 |
| 2017 | NfPfK240 | 4.15 | 4.70 | 9.29 |  | 7.08 | 8.24 | 10.83 |  | 8.87 | 14.00 | 13.12 |
| 2017 | NfPfK120 | 2.92 | 5.15 | 4.88 |  | 7.82 | 9.03 | 10.52 |  | 4.44 | 10.88 | 8.22 |
| 2017 | NfPfK60 | 4.57 | 3.33 | 5.06 |  | 7.15 | 6.78 | 8.89 |  | 3.25 | 9.28 | 6.96 |
| 2017 | N75P20K90 | 3.30 | 2.49 | 3.16 |  | 5.35 | 5.11 | 7.01 |  | 4.22 | 8.22 | 6.35 |
|  |  | P, g m^-2^ | | | | | | | | | | |
| 2017 | N0PfKf | 0.25 | 0.46 | 0.86 |  | 0.23 | 0.50 | 1.13 |  | 0.35 | 1.46 | 1.58 |
| 2017 | NfP0Kf | 0.21 | 0.28 | 0.71 |  | 0.51 | 0.48 | 0.83 |  | 0.35 | 1.08 | 1.00 |
| 2017 | NfPfK0 | 0.19 | 1.24 | 0.87 |  | 0.30 | 1.21 | 1.16 |  | 0.30 | 1.56 | 1.32 |
| 2017 | NfPfK240 | 0.30 | 0.73 | 1.69 |  | 0.37 | 0.80 | 1.70 |  | 0.69 | 2.40 | 3.10 |
| 2017 | NfPfK120 | 0.23 | 0.72 | 0.86 |  | 0.47 | 1.05 | 1.73 |  | 0.36 | 1.77 | 1.74 |
| 2017 | NfPfK60 | 0.33 | 0.50 | 0.91 |  | 0.38 | 0.71 | 1.28 |  | 0.25 | 1.62 | 1.45 |
| 2017 | N75P20K90 | 0.22 | 0.31 | 0.69 |  | 0.27 | 0.46 | 1.09 |  | 0.30 | 1.02 | 1.17 |
|  |  | K, g m^-2^ | | | | | | | | | | |
| 2017 | N0PfKf | 1.32 | 2.55 | 7.31 |  | 0.94 | 4.07 | 9.04 |  | 0.85 | 4.22 | 8.27 |
| 2017 | NfP0Kf | 1.63 | 3.65 | 6.33 |  | 2.72 | 6.28 | 11.17 |  | 1.50 | 11.63 | 8.97 |
| 2017 | NfPfK0 | 0.57 | 4.88 | 4.54 |  | 1.02 | 4.89 | 6.37 |  | 0.44 | 2.53 | 3.67 |
| 2017 | NfPfK240 | 1.58 | 4.58 | 9.75 |  | 1.63 | 6.44 | 14.94 |  | 1.89 | 6.00 | 13.99 |
| 2017 | NfPfK120 | 1.03 | 3.42 | 5.27 |  | 1.64 | 5.00 | 11.33 |  | 0.90 | 5.52 | 7.13 |
| 2017 | NfPfK60 | 1.45 | 2.62 | 4.79 |  | 1.30 | 4.00 | 7.60 |  | 0.50 | 3.25 | 4.67 |
| 2017 | N75P20K90 | 1.24 | 3.11 | 6.20 |  | 1.09 | 3.14 | 9.10 |  | 0.66 | 3.10 | 5.65 |

Table S2. The F-values for factors included in the mixed model ANOVA analysis for N, P and K uptake at final harvest. In these mixed models, year was included as random factor.

| Component | Factors | N uptake | |  | P uptake | |  | K uptake | |
| --- | --- | --- | --- | --- | --- | --- | --- | --- | --- |
|  |  | F value | Significance^1^ |  | F value | Significance^1^ |  | F value | Significance^1^ |
| Leaves | Treatment | 3.9 | *** |  | 3.2 | *** |  | 7.4 | *** |
|  | Location | 13.8 | *** |  | 22.7 | *** |  | 1.4 | NS |
|  | Treatment × Location | 0.8 | NS |  | 0.6 | NS |  | 0.9 | NS |
| Stem | Treatment | 6.8 | *** |  | 9.6 | *** |  | 30.4 | *** |
|  | Location | 82.6 | *** |  | 121.3 | *** |  | 59.9 | *** |
|  | Treatment × Location | 1.4 | NS |  | 2.5 | ** |  | 4.2 | *** |
| Root | Treatment | 13.8 | *** |  | 16.9 | *** |  | 8.0 | NS |
|  | Location | 38.2 | *** |  | 29.1 | NS |  | 1.2 | NS |
|  | Treatment × Location | 0.7 | NS |  | 0.9 | NS |  | 0.3 | NS |

^1^NS, not significant; P<0.05, *; P<0.01, **; P<0.001, ***;


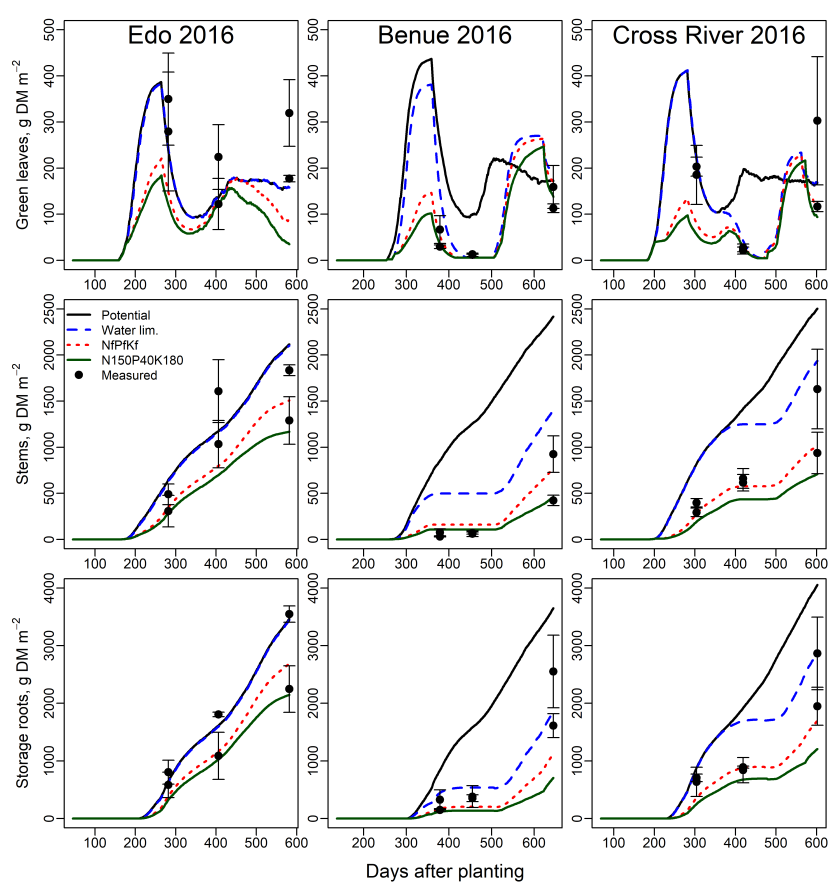


Fig. S5. Observed and simulated green leaves, stems, and storage root yields under water- and nutrient limited conditions from Edo, Cross River and Benue in 2016. Error bars indicate one standard deviation of the observed means.


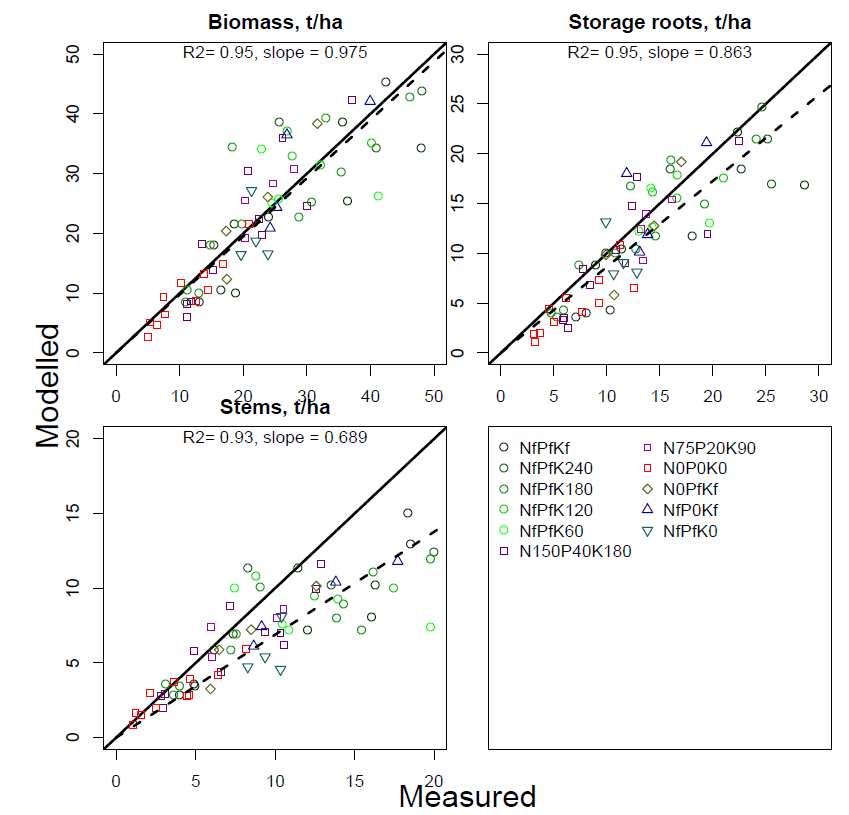


Fig S6. Measured vs modelled values for biomass, storage organs and stems for Cross River and Edo sites in 2016 and 2017. Measured biomass included stems, hanging leaves, petioles, and storage roots. Modelled biomass includes stems, storage roots, fine roots, green leaves and dead leaves.

Table S3. Amounts of leaves, stem, and storage roots at about 4 months after planting (MAP), for cassava grown in Benue, Cross River and Edo. The treatments indicate the amounts applied in kg N, P or K per ha. The highest amounts applied are 300 kg/ha for N and K and 100 kg /ha for P, referred to as the full (f) dose.

| Treatment | Benue | | |  | Cross River | | |  | Edo | | |
| --- | --- | --- | --- | --- | --- | --- | --- | --- | --- | --- | --- |
|  | Leaf | Stem | Roots |  | Leaf | Stem | Roots |  | Leaf | Stem | Roots |
|  | (g m^-2^) | (g m^-2^) | (g m^-2^) |  | (g m^-2^) | (g m^-2^) | (g m^-2^) |  | (g m^-2^) | (g m^-2^) | (g m^-2^) |
|  |  |  |  |  |  | 2016 |  |  |  |  |  |
| Control | 13.5 | 13.6 | 89.1 |  | 71.6 | 104.8 | 320.8 |  | 97.0 | 121.1 | 311.4 |
| N0PfKf | 22.5 | 23.5 | 164.3 |  | 82.9 | 135.2 | 335.2 |  | 153.1 | 180.7 | 358.2 |
| NfP0Kf | 30.6 | 30.5 | 176.1 |  | 91.8 | 141.1 | 232.3 |  | 280.5 | 307.8 | 491.6 |
| NfPfK0 | 41.4 | 46.5 | 204.8 |  | 203.3 | 433.6 | 539.4 |  | 195.1 | 211.0 | 372.5 |
| NfPfKf | 66.7 | 77.2 | 326.0 |  | 203.1 | 398.0 | 706.2 |  | 349.7 | 489.5 | 805.1 |
| NfPfK240 | 59.1 | 54.1 | 299.6 |  | 170.6 | 388.4 | 463.7 |  | 335.5 | 374.7 | 515.2 |
| NfPfK180 | 61.0 | 60.0 | 234.0 |  | 203.3 | 360.1 | 528.5 |  | 332.0 | 310.4 | 474.2 |
| NfPfK120 | 52.1 | 52.8 | 232.6 |  | 217.5 | 482.9 | 649.7 |  | 282.1 | 380.3 | 613.4 |
| NfPfK60 | 40.4 | 48.9 | 137.6 |  | 169.9 | 419.1 | 452.2 |  | 270.8 | 327.0 | 454.4 |
| N150P40K180 | 29.6 | 30.3 | 149.3 |  | 185.3 | 293.3 | 635.3 |  | 279.3 | 307.3 | 584.7 |
| N75P20K90 | 28.1 | 32.0 | 180.9 |  | 131.0 | 262.9 | 583.4 |  | 290.0 | 321.6 | 705.7 |
|  |  |  |  |  |  | 2017 |  |  |  |  |  |
| Control | 60.3 | 96.4 | 290.5 |  |  |  |  |  | 115.1 | 155.7 | 370.7 |
| N0PfKf | 50.1 | 101.5 | 332.7 |  |  |  |  |  | 117.6 | 134.6 | 418.2 |
| NfP0Kf | 82.6 | 116.1 | 311.6 |  |  |  |  |  | 287.6 | 366.5 | 773.6 |
| NfPfK0 | 97.8 | 205.8 | 449.5 |  |  |  |  |  | 132.9 | 230.7 | 383.5 |
| NfPfKf | 89.9 | 154.1 | 459.8 |  |  |  |  |  | 349.7 | 495.3 | 1030.3 |
| NfPfK240 | 115.6 | 215.6 | 555.9 |  |  |  |  |  | 243.7 | 361.1 | 676.7 |
| NfPfK180 | 110.7 | 192.7 | 375.5 |  |  |  |  |  | 311.1 | 398.7 | 591.3 |
| NfPfK120 | 122.2 | 221.9 | 509.6 |  |  |  |  |  | 277.1 | 422.4 | 809.9 |
| NfPfK60 | 84.6 | 144.4 | 366.9 |  |  |  |  |  | 222.7 | 358.2 | 652.9 |
| N150P40K180 | 92.1 | 163.6 | 403.5 |  |  |  |  |  | 239.6 | 281.4 | 597.0 |
| N75P20K90 | 72.0 | 122.9 | 326.2 |  |  |  |  |  | 206.9 | 273.0 | 605.3 |


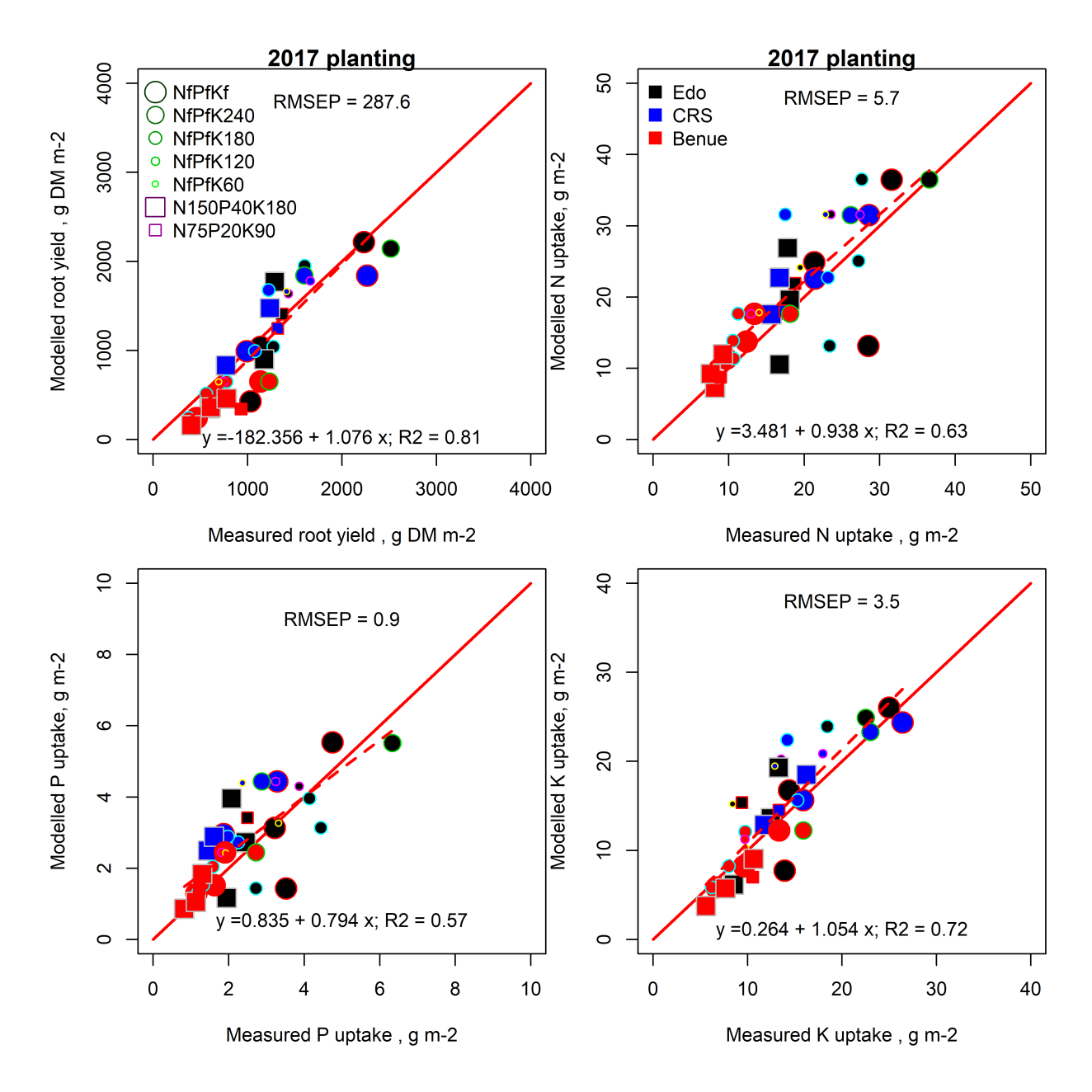


Fig. S7. Observed and simulated storage roots, N, P and K uptakes for the selected treatments, excluding treatments used to estimate soil N, P and K supply from Edo, Cross River (CRS) and Benue in 2017 at final harvest. The solid line is the 1:1 line, while the dashed line is the best fit regression.

Table S4. Amounts of leaves, stem, and storage roots at about 8 months after planting (MAP), for cassava grown in Benue, Cross River and Edo. The treatments indicate the amounts applied in kg N, P or K per ha. The highest amounts applied are 300 kg/ha for N and K and 100 kg /ha for P, referred to as the full (f) dose.

|  | Benue | | |  | Cross River | | | |  | | Edo | | |
| --- | --- | --- | --- | --- | --- | --- | --- | --- | --- | --- | --- | --- | --- |
|  | Leaf | Stem | Roots |  | Leaf | Stem | Roots |  | | Leaf | | Stem | Roots |
|  | (g m^-2^) | (g m^-2^) | (g m^-2^) |  | (g m^-2^) | (g m^-2^) | (g m^-2^) |  | | (g m^-2^) | | (g m^-2^) | (g m^-2^) |
|  | 2016 | | | | | | | | | | | | |
| Control | 4.1 | 12.7 | 116.1 |  | 10.2 | 249.7 | 503.3 |  | | 42.9 | | 361.2 | 615.1 |
| N0PfKf | 7.2 | 30.7 | 207.1 |  | 17.6 | 344.5 | 680.1 |  | | 116.6 | | 757.6 | 1023.0 |
| NfP0Kf | 10.1 | 44.0 | 182.1 |  | 52.5 | 509.9 | 515.1 |  | | 246.2 | | 1421.1 | 1396.5 |
| NfPfK0 | 12.1 | 66.5 | 268.4 |  | 39.1 | 855.6 | 872.5 |  | | 143.9 | | 659.8 | 698.0 |
| NfPfKf | 11.9 | 62.0 | 380.8 |  | 27.0 | 615.8 | 891.8 |  | | 224.0 | | 1607.3 | 1806.7 |
| NfPfK240 | 11.3 | 66.1 | 226.8 |  | 19.7 | 767.8 | 534.7 |  | | 147.0 | | 1241.8 | 1170.4 |
| NfPfK180 | 11.3 | 64.5 | 261.8 |  | 21.4 | 721.2 | 734.6 |  | | 227.3 | | 1385.5 | 1457.9 |
| NfPfK120 | 12.6 | 65.2 | 234.3 |  | 31.0 | 870.1 | 734.2 |  | | 148.5 | | 934.5 | 1079.1 |
| NfPfK60 | 16.3 | 68.7 | 268.6 |  | 37.7 | 810.7 | 737.7 |  | | 209.8 | | 1265.0 | 1291.1 |
| N150P40K180 | 13.2 | 69.8 | 352.8 |  | 21.3 | 661.7 | 839.9 |  | | 122.0 | | 1033.7 | 1088.9 |
| N75P20K90 | 9.0 | 47.1 | 263.4 |  | 10.6 | 435.9 | 834.2 |  | | 72.1 | | 610.5 | 845.2 |
|  | 2017 | | | | | | | | | | | | |
| Control | 18.8 | 127.4 | 398.5 |  | 74.8 | 211.9 | 456.4 |  | | 44.4 | | 447.9 | 766.1 |
| N0PfKf | 16.3 | 144.7 | 517.2 |  | 90.6 | 265.9 | 471.9 |  | | 53.8 | | 567.7 | 1056.2 |
| NfP0Kf | 24.4 | 166.4 | 503.4 |  | 85.5 | 384.9 | 661.8 |  | | 68.8 | | 766.9 | 943.4 |
| NfPfK0 | 40.7 | 360.9 | 661.9 |  | 79.9 | 320.7 | 545.8 |  | | 61.6 | | 718.3 | 740.9 |
| NfPfKf | 49.4 | 286.3 | 668.5 |  | 130.1 | 735.3 | 990.0 |  | | 54.2 | | 1203.7 | 1138.6 |
| NfPfK240 | 26.1 | 321.0 | 606.9 |  | 162.1 | 568.4 | 818.3 |  | | 67.1 | | 1313.4 | 1160.2 |
| NfPfK180 | 26.6 | 379.2 | 562.5 |  | 139.5 | 756.1 | 1078.9 |  | | 54.5 | | 1543.3 | 1274.5 |
| NfPfK120 | 39.3 | 1397.9 | 666.5 |  | 78.6 | 491.6 | 656.9 |  | | 61.7 | | 1188.3 | 1196.1 |
| NfPfK60 | 26.1 | 215.9 | 460.5 |  | 89.3 | 521.0 | 674.0 |  | | 32.5 | | 754.0 | 816.7 |
| N150P40K180 | 17.3 | 248.4 | 611.0 |  | 93.8 | 490.2 | 771.8 |  | | 65.4 | | 1057.7 | 1175.1 |
| N75P20K90 | 22.2 | 188.1 | 483.9 |  | 80.7 | 328.5 | 697.6 |  | | 62.6 | | 766.1 | 1127.4 |

Table S5. Amounts of leaves, stem, and storage roots at final harvest, for cassava grown in Benue, Cross River and Edo. The treatments indicate the amounts applied in kg N, P or K per ha. The highest amounts applied are 300 kg/ha for N and K and 100 kg /ha for P, referred to as the full (f) dose.

|  | Benue | | |  | Cross River | | | |  | | Edo | | |
| --- | --- | --- | --- | --- | --- | --- | --- | --- | --- | --- | --- | --- | --- |
|  | Leaf | Stem | Roots |  | Leaf | Stem | Roots |  | | Leaf | | Stem | Roots |
|  | (g m^-2^) | (g m^-2^) | (g m^-2^) |  | (g m^-2^) | (g m^-2^) | (g m^-2^) |  | | (g m^-2^) | | (g m^-2^) | (g m^-2^) |
|  | 2016 | | | | | | | | | | | | |
| Control | 45.4 | 226.4 | 858.7 |  | 63.3 | 460.0 | 926.0 |  | | 153.8 | | 818.1 | 1121.1 |
| N0PfKf | 79.8 | 270.7 | 1004.1 |  | 79.1 | 593.1 | 1067.5 |  | | 201.4 | | 1259.4 | 1702.0 |
| NfP0Kf | 138.7 | 523.6 | 1003.3 |  | 248.1 | 864.9 | 1308.2 |  | | 282.4 | | 1771.0 | 1939.9 |
| NfPfK0 | 136.0 | 735.3 | 1931.5 |  | 105.4 | 936.6 | 1155.9 |  | | 68.9 | | 1033.9 | 1282.9 |
| NfPfKf | 159.0 | 925.6 | 2551.5 |  | 302.7 | 1630.1 | 2866.4 |  | | 319.4 | | 1834.6 | 3546.9 |
| NfPfK240 | 126.0 | 595.5 | 1451.8 |  | 182.6 | 1352.2 | 2553.5 |  | | 329.6 | | 2558.1 | 2462.8 |
| NfPfK180 | 141.3 | 779.9 | 1928.2 |  | 188.1 | 1430.3 | 1922.5 |  | | 232.0 | | 1977.0 | 2410.3 |
| NfPfK120 | 158.0 | 775.6 | 1951.6 |  | 157.6 | 1394.7 | 1659.7 |  | | 169.9 | | 1744.6 | 2100.0 |
| NfPfK60 | 127.9 | 551.2 | 1728.4 |  | 82.9 | 1043.6 | 1426.7 |  | | 176.5 | | 1976.7 | 1969.3 |
| N150P40K180 | 112.8 | 423.5 | 1613.7 |  | 116.6 | 938.1 | 1949.4 |  | | 177.2 | | 1290.5 | 2247.4 |
| N75P20K90 | 99.0 | 320.7 | 1100.2 |  | 72.8 | 603.6 | 1342.5 |  | | 135.6 | | 1051.9 | 1611.0 |
|  | 2017 | | | | | | | | | | | | |
| Control | 93.0 | 293.4 | 587.9 |  | 83.1 | 466.3 | 1252.6 |  | | 114.6 | | 642.4 | 922.9 |
| N0PfKf | 101.5 | 329.6 | 885.7 |  | 90.5 | 647.4 | 991.6 |  | | 90.0 | | 849.1 | 1445.6 |
| NfP0Kf | 100.4 | 416.8 | 761.3 |  | 227.8 | 914.4 | 1383.7 |  | | 116.9 | | 1381.5 | 1186.7 |
| NfPfK0 | 65.9 | 869.0 | 765.0 |  | 98.4 | 1040.4 | 989.6 |  | | 71.7 | | 827.7 | 1063.1 |
| NfPfKf | 109.9 | 466.9 | 1135.8 |  | 147.6 | 1143.1 | 2267.0 |  | | 157.6 | | 1852.3 | 2233.0 |
| NfPfK240 | 107.5 | 486.5 | 1231.6 |  | 137.6 | 828.7 | 1599.0 |  | | 179.6 | | 1999.2 | 2516.6 |
| NfPfK180 | 64.4 | 427.3 | 777.6 |  | 156.0 | 905.3 | 1222.2 |  | | 75.0 | | 1616.3 | 1604.9 |
| NfPfK120 | 87.4 | 439.7 | 744.9 |  | 150.3 | 878.4 | 1662.9 |  | | 90.4 | | 1248.4 | 1432.3 |
| NfPfK60 | 119.1 | 382.0 | 693.4 |  | 129.4 | 744.0 | 1413.9 |  | | 64.2 | | 1086.0 | 1300.4 |
| N150P40K180 | 90.0 | 360.4 | 779.1 |  | 118.0 | 717.6 | 1236.4 |  | | 72.2 | | 1255.9 | 1287.7 |
| N75P20K90 | 111.4 | 416.7 | 931.3 |  | 105.1 | 594.1 | 1320.1 |  | | 90.6 | | 1012.7 | 1367.7 |

Table S6. Analysis of variance in leave, stem and storage root biomass for factors included in the mixed model in Tables S3, S4 and S5, with year as random factor.

|  |  | 4 MAP | |  | 8MAP | |  | Final harvest | |
| --- | --- | --- | --- | --- | --- | --- | --- | --- | --- |
| Component | Factor | F value | P value |  | P value | P value |  | P value | P value |
| Leaves | Treatment | 6.7 | 0.001 |  | NS | NS |  | 3.6 | 0.01 |
|  | Location | 149.5 | 0.001 |  | 0.001 | 0.001 |  | 6.5 | 0.01 |
|  | Treatment × Location | 1.9 | NS |  | NS | NS |  | 0.9 | NS |
|  |  |  |  |  |  |  |  |  |  |
| Stem | Treatment | 13.5 | 0.001 |  | 0.001 | 0.001 |  | 14.8 | 0.001 |
|  | Location | 161.5 | 0.001 |  | 0.001 | 0.001 |  | 150.1 | 0.001 |
|  | Treatment × Location | 3.4 | 0.01 |  | NS | NS |  | 3.0 | 0.01 |
|  |  |  |  |  |  |  |  |  |  |
| Storage roots | Treatment | 9.9 | 0.001 |  | 0.01 | 0.01 |  | 12.2 | 0.001 |
|  | Location | 102.8 | 0.001 |  | 0.001 | 0.001 |  | 19.2 | 0.001 |
|  | Treatment × Location | 2.6 | 0.01 |  | NS | NS |  | 0.8 | NS |

Table S7. Parameter values for the LINTUL-CASSAVA-NPK model

| Programming codes | Meaning | Units | Value | Source |
| --- | --- | --- | --- | --- |
| FASTRANSLSO | Fraction of senesced leaf weight translocated to storage roots before the shedding of the life | - | 0.65 | Adiele et al. (2021b) |
| FRACTLLFENHSH | Fraction of leaf life at which enhanced shedding can be induced | - | 0.85 | Ezui et al., (2018) |
| WSOREDISTFRACM | Maximum fraction of dry matter redistribution from storage roots for the formation of new leaves | - | 0.05 | Ezui et al., (2018) |
| RECOV | Fraction of critical soil water content above which the crop recovers from drought | - | 0.7 | Ezui et al., (2018) |
| WCUTTINGMINF RAC | Fraction of the stem cutting weight that remains after being used for the plant's growth | - | 0.15 | Ezui et al., (2018) |
| *k* | Light extinction coefficient | - | 0.67 | Adiele et al. (2021b) |
| LAICR | Critical leaf area index beyond which growth rate is diminished by shading | m^2^ leaf m^−2^ soil | 3.5 | Ezui et al., (2018) |
| LAIEXPOEND | Maximum leaf area index where exponential growth phase ends | m^2^ leaf m^−2^ soil | 0.75 | Ezui et al., (2018) |
| LAI_MIN | Minimum leaf area index where dormancy may start | m^2^ leaf m^−2^ soil | 0.09 | Adiele et al. (2021b) |
| LUE_OPT | Light use efficiency at optimum growing conditions | g DM MJ IPAR^−1^ | 2.76 | Adiele et al. (2021b) |
| RDRSDM | Maximum relative death rate of leaves due to severe drought | d^−1^ | 0.09 | Ezui et al., (2018) |
| RDRSHM | Relative death rate of leaves due to shade | d^−1^ | 0.09 | Ezui et al., (2018) |
| RDRWCUTTING | Relative decrease rate of cutting weight | d^−1^ | 0.017 | Alves (2002) |
| RGRL | Relative growth rate of leaf area of young leaves in the exponential phase per degree centigrade | °C d^−1^ | 0.003 | Ezui et al., (2018) |
| RRREDISTSO | Relative rate of redistribution of dry matter from storage roots to leaves | d^−1^ | 0.01 | Ezui et al., (2018) |
| RRDMAX | Maximum rate of increase in rooting depth in a homogeneous deep soil | m d^−1^ | 0.022 | Matthews and Hunt (1994) |
| SO2LV | Conversion ratio of storage organs dry matter to leaf dry matter | g leaf DM g^−1^ storage root DM | 0.8 | Ezui et al., (2018) |
| SLA_MAX | Maximum specific leaf area | m2 leaf g^−1^ leaf | 0.03 | Adiele et al. (2021b) |
| TBASE | Base temperature below which no crop development takes place | °C | 15 | Alves (2002) |
| TSUMBR_ONE | Temperature sum accumulation to first branching | °C d | 776 | Gutierrez et al. (1988) |
| TSUMSOBULKINIT | Temperature sum accumulation to the start of storage roots bulking | °C d | 529 | El-Sharkawy (2003) |
| DELREDIST | Delay for redistribution of dry matter | °C d | 12 | Ezui et al., 2018 |
| TSUMREDISTMAX | Maximum temperature sum accumulation to indicate the duration of dry matter redistribution | °C d | 144 | Ezui et al., (2018) |
| EMERGTSUM_AVE | Average temperature sum to be reached from planting to the moment of sprouting | °C d | 170 | Alves (2002) |
| TSUMLA_MIN | Temperature sum accumulation to achieve a minimum leaf area index before the end of its exponential growth phase | °C d | 180 | Alves (2002) |
| TSUMLLIFE | Temperature sum indicating the leaf developing time from sprouting of a new leaf to its shedding | °C d | 1500 | Alves (2002) |
| WCUTTINGUNIT | Average weight per cutting | g m^−2^ | 14 | van Heemst (1988) |
| WLVGNEWM | Maximum amount of new leaves weight produced in the redistribution phase | g DM m^−2^ | 10 | Ezui et al., 2018 |
| CmaxL_N_ | Maximum N concentration in the leaves | g kg N kg^-1^ DM | 0.0550 | Adiele et al. (2021a) |
| CmaxL_P_ | Maximum P concentration in the leaves | g kg P kg^-1^ DM | 0.0044 | Adiele et al. (2021a) |
| CmaxL_K_ | Maximum K concentration in the leaves | g kg K kg^-1^ DM | 0.0211 | Adiele et al. (2021a) |
| CmaxST_N_ | Maximum N concentration in the stems | g kg N kg^-1^ DM | 0.0116 | Adiele et al. (2021 a) |
| CmaxST_P_ | Maximum P concentration in the stems | g kg P kg^-1^ DM | 0.0026 | Adiele et al. (2021a) |
| CmaxST_K_ | Maximum K concentration in the stems | g kg K kg^-1^ DM | 0.0126 | Adiele et al. (2021a) |
| CmaxSO_N_ | Maximum N concentration in the storage roots | g kg N kg^-1^ DM | 0.0158 | Adiele et al. (2021a) |
| CmaxSO_P_ | Maximum P concentration in the storage roots | g kg P kg^-1^ DM | 0.0022 | Adiele et al. (2021a) |
| CmaxSO_K_ | Maximum K concentration in the storage roots | g kg K kg^-1^ DM | 0.0125 | Adiele et al. (2021a) |
| CminL_N_ | Minimum N concentration in the leaves | g kg N kg^-1^ DM | 0.0260 | Adiele et al. (2021a) |
| CminL_P_ | Minimum P concentration in the leaves | g kg P kg^-1^ DM | 0.0018 | Adiele et al. (2021a) |
| CminL_K_ | Minimum K concentration in the leaves | g kg K kg^-1^ DM | 0.0041 | Adiele et al. (2021a) |
| CminST_N_ | Minimum N concentration in the stems | g kg N kg^-1^ DM | 0.0043 | Adiele et al. (2021a) |
| CminST_P_ | Minimum P concentration in the stems | g kg P kg^-1^ DM | 0.0004 | Adiele et al. (2021a) |
| CminST_K_ | Minimum K concentration in the stems | g kg K kg^-1^ DM | 0.0015 | Adiele et al. (2021a) |
| CminSO_N_ | Minimum N concentration in the storage roots | g kg N kg^-1^ DM | 0.0021 | Adiele et al. (2021a) |
| CminSO_P_ | Minimum P concentration in the storage roots | g kg P kg^-1^ DM | 0.0005 | Adiele et al. (2021a) |
| CminSO_K_ | Minimum K concentration in the storage roots | g kg K kg^-1^ DM | 0.0024 | Adiele et al. (2021a) |

Table S8. Nutrient application rates per treatment, (f) represents full rate of the optimized nutrient and K60, K120, K180, K240, were varied rates of K at 60, 120, 180, and 240 kg ha^-1^

|  | Treatment | Nitrogen | Phosphorus | Potassium |
| --- | --- | --- | --- | --- |
|  |  | (kg N ha^-1^) | (kg P ha^-1^) | (kg K ha^-1^) |
| 1. | Control | 0 | 0 | 0 |
| 2. | N0PfKf | 0 | 100 | 300 |
| 3. | NfP0Kf | 300 | 0 | 300 |
| 4. | NfPfK0 | 300 | 100 | 0 |
| 5. | NfPfK60 | 300 | 100 | 60 |
| 6. | NfPfK120 | 300 | 100 | 120 |
| 7. | NfPfK180 | 300 | 100 | 180 |
| 8. | NfPfK240 | 300 | 100 | 240 |
| 9. | N150P40K180 | 150 | 40 | 180 |
| 10. | N75P20K90 | 75 | 20 | 90 |
| 11. | NfPfKf | 300 | 100 | 300 |
